# Beyond Pain Relief: A Review on Cannabidiol Potential in Medical Therapies

**DOI:** 10.3390/ph16020155

**Published:** 2023-01-20

**Authors:** Mariana Luz-Veiga, João Azevedo-Silva, João C. Fernandes

**Affiliations:** 1 CBQF—Centro de Biotecnologia e Química Fina—Laboratório Associado, Escola Superior de Biotecnologia, Universidade Católica Portuguesa, 4169-005 Porto, Portugal; 2 Amyris Bio Products Portugal, Unipessoal Lda, 4169-005 Porto, Portugal

**Keywords:** cannabidiol, cannabinoids, cancer, neurologic conditions, auto immune diseases

## Abstract

The phytocannabinoid cannabidiol (CBD) is receiving increasing attention due to its pharmacological properties. Although CBD is extracted from *Cannabis sativa*, it lacks the psychoactive effects of Δ9-tetrahydrocannabinol (THC) and has become an attractive compound for pharmacological uses due to its anti-inflammatory, antioxidant, anticonvulsant, and anxiolytic potential. The molecular mechanisms involved in CBD’s biological effects are not limited to its interaction with classical cannabinoid receptors, exerting anti-inflammatory or pain-relief effects. Several pieces of evidence demonstrate that CBD interacts with other receptors and cellular signaling cascades, which further support CBD’s therapeutic potential beyond pain management. In this review, we take a closer look at the molecular mechanisms of CBD and its potential therapeutic application in the context of cancer, neurodegeneration, and autoimmune diseases.

## 1. Introduction

**Figure 1:**
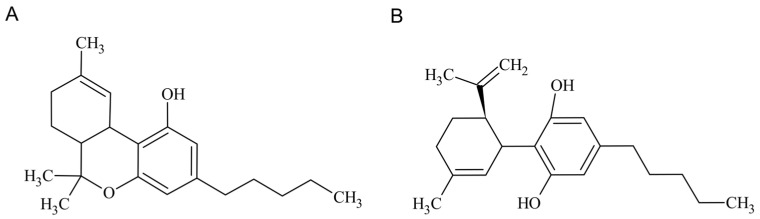
THC (**A**) and CBD (**B**) chemical structures.

Cannabidiol (CBD) is one of the main pharmacologically-active cannabinoids, known for its several biological activities, including anti-inflammatory, antioxidant, anticonvulsant, and anxiolytic properties [1,2,3,4]. Although it possesses psychoactive activity, this phytocannabinoid extracted from *Cannabis sativa* does not provoke any intoxicating effects, unlike its counterpart Δ9-tetrahydrocannabinol (THC) [5]. Through decarboxylation reactions that occur upon heating, tetrahydrocannabinolic acid-A (THCA-A) and cannabidiolic acid (CBDA) are converted into THC and CBD, respectively [6]. Chemically, CBD is a terpenophenol molecule containing a cyclohexene ring, a phenolic ring, and a pentyl side chain, as shown in Figure 1 [1]. Regarding chemical stability, cannabinoids have been regarded as highly unstable molecules, being photolabile, thermolabile, and sensitive to oxidation, with cannabis oil preparations showing higher thermal stability [7,8,9]. 

Although interest in research regarding cannabinoids has increased in the past decades, concerns remain regarding their use, mainly because this compound is extracted from the *Cannabis* plant [10,11]. Legal regulations concerning the cultivation of *Cannabis* for use for therapeutic purposes vary from country to country and the cultivation of hemp has an increasing environmental impact and leads to contamination with pesticides and heavy metals [12,13,14,15]. The World Anti-drug Doping Agency (WADA) has placed cannabis on its list of banned compounds, with its presence in athletes being monitored by the measurement of THC in urine [16]. CBD, on the other hand, has been suggested as potentially beneficial to athletes, since it is not on the list of WADA’s banned compounds and could be used to manage pain, inflammation, and swelling associated with injuries, as shown in clinical and preclinical models [17,18,19]. In addition, pure THC has been described as a potentially dangerous drug. Efforts have been made to circumvent these problems, with synthetic CBD being produced entirely without resourcing to natural cannabinoids [20]. 

The therapeutic potential of CBD has been evaluated in cardiovascular, neurodegenerative, and metabolic conditions, as well as for cancer [1,2,21,22,23,24,25]. These pathologies are usually associated with oxidative stress and inflammation [1]. Several studies have described CBD as immune suppressive and anti-inflammatory, with its overall mechanism of action involving the direct suppression of target cells (e.g., effector T cells and microglial cells) by suppressing kinase cascades and different transcription factors [2,26,27,28].

Taking into account the potential benefits of CBD and other cannabinoids for several diseases with an inflammation phenotype, this review aims to discuss the therapeutic effect of CBD, considering its mechanism of action and incorporating clinical data whenever possible.

## 2. The Endocannabinoid System

Phytocannabinoids exert a strong effect on the endocannabinoid system (ECS), which is an important molecular system consisting of cannabinoid receptors (CB_1_ and CB_2_). Cannabinoid receptors are expressed in virtually all tissues; endogenous cannabinoids (endocannabinoids); and the enzymes responsible for synthesizing and breaking down endocannabinoids, namely fatty acid amide hydrolase (FAAH) and monoacylglycerol lipase (MAGL) [29]. Although initially described as an inverse agonist for both receptors, recent studies have demonstrated that CBD has a low affinity for the cannabinoid receptors, not targeting them directly [30]. However, CBD can antagonize them in the presence of THC and can also act as a non-competitive negative allosteric modulator of the CB_1_ receptor [30].

The ECS has been implicated in various biological processes, including, but not limited to, the central nervous system (CNS) development, synaptic plasticity, and the response to endogenous and environmental insults, including pain sensation and itch [31,32]. As such, the modulation of ECS activity has demonstrated the potential to ameliorate and treat several pathologies, including cancer, as well as inflammatory, neurodegenerative, and cardiovascular disorders, becoming an increasingly popular target for pharmacotherapy [33,34,35,36]. For instance, CB_2_ receptor activation has been regarded as leading to anti-inflammatory effects, being a potential target in conditions such as rheumatoid arthritis, atopic dermatitis, atherosclerosis, and inflammatory bowel disease [37,38]. Endocannabinoids are retrograde neurotransmitters, and their synthesis in the brain is mainly stimulated by an intracellular increase in Ca^2+^ [39,40]. Some endocannabinoids which have been described to date include 2-arachidonoyl glycerol (2-AG), N-arachidonoylethanolamine (AEA or anandamide), 2-arachidonyl glyceryl ether (noladin ether), O-arachidonoylethanolamine (virodhamine), and N-arachidonoyl-dopamine (NADA), with 2-AG and AEA being the most extensively studied [29]. Their physiological effects are mostly exerted through CB_1_ and CB_2_, with 2-AG being a strong agonist for both CB_1_ and CB_2_ receptors, while AEA is a low-efficacy agonist for CB_1_ and has a very low agonist efficacy for CB_2_ [38,41,42,43]. Additionally, FAAH and MAGL are responsible for the degradation of AEA and 2-AG, respectively, playing a fundamental role in controlling their tissue levels [44]. Reports have shown CBD’s ability to inhibit FAAH, leading to an increase in AEA levels, which may have therapeutic potential, particularly for pain and anxiety disorders [45,46]. Moreover, Habib, et al. [47] observed that depletion of the FAAH gene led to higher endocannabinoid signaling and consequent pain insensitivity. Cravatt, et al. [48] reported how genetic invalidation of FAAH led to an increase in endocannabinoid levels in tissues. On a similar note, Schlosburg, et al. [49] demonstrated how the deletion of MAGL strongly increased the levels of 2-AG, which has been implicated in the recruitment and migration of dendritic cells, B and T cells, and monocytes in a CB_2_-dependent manner [43].

## 3. CBD Interaction with Other Cellular Receptors

CBD has been reported as interacting with several receptors, being able to activate, modulate, and inhibit different pathways as a result. In Table 1, information regarding the CBD dose of each study is presented. Although some in vitro studies have shown the effect of CBD in a receptor/target, caution must be taken due to the physiological levels of CBD which are permitted. CBD activates 5-HT_1A_ serotonergic and vanilloid receptors TRPV1-2, TRPV1-3, and TRPV4, and acts as an inverse agonist to G protein-coupled receptor (GPR) 3, GPR6, and GPR12, while being an antagonist for GPR55 [50,51,52,53,54,55]. These interactions may present promising therapeutic potential, since these receptors are involved in a vast array of pathologies, ranging from neurological conditions such as epilepsy and anxiety, to diabetes, cancer, and immunological disorders [56,57,58,59]. Additionally, CBD is an agonist for peroxisome proliferator-activated receptor gamma (PPAR-γ), up-regulating these receptors [60]. PPAR-γ activation prevents the NF-κb signaling pathway, the incorrect regulation of which has been associated with inflammatory conditions and cancer [61,62,63]. CBD also affects calcium levels, activating mitochondrial complexes I, II, II-III, and IV, and, consequently, regulating mitochondria Ca^2+^ stores and blocking voltage-gated T-type calcium channels [64,65,66]. Moreover, CBD inhibits equilibrative nucleoside transporters (ENT1), reducing adenosine reuptake [64].

Synthetic cannabinoids have also been described as possessing bioactivity. Mascal, Hafezi, Wang, Hu, Serra, Dallas and Spencer [11] reported how 8,9-dihydrocannabidiol, a CBD synthetic analogue, showed similar antiseizure effects in rats, reducing the severity and number of seizures. Another study using synthetic CBD, originating exclusively from pharmaceutical-grade substances, demonstrated its efficacy and tolerance in epilepsy treatment to be comparable to plant-derived CBD [93]. Other compounds have been synthesized from CBD, resulting in a similar therapeutic potential. For instance, HU-331 is a quinone anticarcinogenic drug and HU-320 has strong anti-inflammatory and immunosuppressive properties that demonstrate no psychoactive effects [94,95]. On a similar note, synthetic CB_2_ agonists have been studied to achieve an effect similar to CBD, with a few animal studies describing JWH 133’s potential anti-cancer, anti-inflammatory, and neuroprotective effects [96,97,98,99]. The antagonist AM630 has been discussed as a CB_2_ receptor protean ligand, with potential therapeutic interest owing to its role as a CB_2_ receptor inverse agonist [100,101,102]. SR144528 is also a CB_2_ receptor-selective antagonist/inverse agonist which is widely used in cannabinoid research [102]. Currently, there are more options on the market to mimic THC than CBD, including nabilone, HU-210, and dexanabinol [6,103]. Nabilone is used as an antiemetic and for chronic pain management, and dexanabinol has been associated with neuroprotective effects [104,105]. These molecules retain THC’s ring structures and oxygen atoms [6].

## 4. CBD’s Anti-Inflammatory and Antioxidant Capacity: From Epilepsy to Depression

Neurodegeneration is a progressive loss of structure, function, and/or death of neurons and neuronal structures, and is the basic process for the development of neurodegenerative conditions. It has been shown that most of the underlying mechanisms common to many neurodegenerative conditions are similar in function, involving the aggregation of misfolded proteins, mitochondrial dysfunction, and oxidative stress [106,107,108,109,110]. Neurodegenerative diseases such as PD, AD, and multiple sclerosis, as well as anxiety and depression, share these imbalances. As such, research focusing on neuroprotection usually targets these imbalances, as well as inflammation and excitotoxicity, which, together, aggravate and contribute to neurodegeneration [106,107].

CBD has been described as exerting neuroprotective effects, and, although several preclinical studies have linked this potential to its anti-inflammatory and antioxidant properties, the underlying mechanisms have yet to be elucidated. Although inflammation is an active defense response to harmful stimuli, it can lead to cellular and tissue damage. Neuroinflammation is present in pathologies such as multiple sclerosis, ischemia, and AD [107]. CBD has been demonstrated to exert anti-inflammatory properties through the activation of the CB2 receptor, which leads to a decrease in ROS and TNF-α levels [37]. Additionally, peroxisome proliferator-activated receptors (PPARs), which are associated with inflammatory responses, have been shown to be activated by CBD; more specifically, the isoform PPARγ, which has been detected in neurons and astrocytes and is often involved in the modulation of inflammation [60,63,111]. PPARγ activation by CBD contributes to an anti-inflammatory response, since this receptor interacts directly with NF-kB, inhibiting the transcription of pro-inflammatory genes (i.e., iNOS and COX-2) [1,111]. Moreover, CBD stimulates the biosynthesis of AEA and 2-AG, which are agonists of the PPARγ receptor [112,113].

Oxidative stress arises when the antioxidant protection systems of the body are unable to contain the production of reactive oxygen species and reactive nitrogen species (ROS/RNS) [114]. The consequent accumulation of these compounds leads to the oxidation of biologically-relevant molecules such as proteins, consequently altering metabolic pathways and disturbing the maintenance of homeostatic balance [115]. ROS production tends to aggravate neurodegenerative conditions [116]. Moreover, neurons have weak antioxidant defenses, namely poor catalase activity and moderate superoxide dismutase and glutathione peroxidase activities; however, they have a high oxygen demand, making them highly susceptible to oxidative stress [117,118].

CBD has demonstrated antioxidant capacity, either by directly affecting components of the redox system or by acting indirectly through the activation of different receptors and modulation of certain molecules’ levels. For instance, CBD can activate transient vanilloid receptors (TRPV) such as TRPV2, and is an inverse agonist of GPR3, GPR6, and GPR12, which directly or indirectly influence oxidative stress [1,50,84]. PPARγ has been shown to interact with the transcription factor erythroid 2-related factor 2 (Nrf-2), which controls gene expression associated with oxidative stress [119,120]. As CBD is an agonist of this receptor, this contributes to its potential antioxidant capacity [1].

Besides the aforementioned properties, CBD possesses other beneficial pharmacological effects, including anticonvulsant, anxiolytic, antipsychotic, and antidepressant properties, thus validating the potential therapeutic features of CBD [1,79,120,121,122,123].

### 4.1. CBD in Epilepsy

The effect of CBD has been tested through clinical trials on some conditions, including epilepsy and PD, with promising results [22,124,125]. Nevertheless, further testing is required to confirm the efficacy of CBD. Owing to its antiseizure properties, CBD has been proposed for the treatment of epilepsy, which is a neurological condition characterized by abnormal brain activity that results in seizures with varying degrees of gravity. Epidiolex is a cannabidiol oral solution which was approved in 2018 by the Food and Drug Administration (FDA) in the USA and in 2019 by the European Medicines Agency (EMA) in Europe. This drug is used to treat seizures associated with Lennox–Gastaut and Dravet syndrome, which are severe forms of epilepsy that mostly affect children [126]. The mechanisms underlying CBD action in epilepsy are still being researched and debated. Its effects are thought to be associated with the antagonism of GPR55 and the consequent inhibition of intracellular calcium release, reducing neuronal hyperexcitability, which could be seen as an antiepileptic feature [4]. Nevertheless, more evidence is needed to support this mechanism of action [25,120,126]. CBD is also a partial agonist of serotonin 1A and 2A (5-HT_1A_ and 5-HT_2A_) receptors, with some authors speculating that this affinity sustains its anticonvulsant effect [126]. As such, targeting these receptors could be a valid therapeutic option due to their regulatory action on neuronal depolarization, although their role in epilepsy is still unclear [125,127]. Nevertheless, Pelz, Schoolcraft, Larson, Spring and López [56] reported that, although CBD decreased seizures in rats, its mechanism of action does not seem to be mediated by these receptors. On a similar note, reports have shown CBD to cause a desensitization of TRPV1 channels, also leading to a decrease in neuronal hyperactivity due to reducing extracellular calcium influx [128]. However, some authors have argued that it is not possible to attribute CBD’s antiepileptic mechanism to its affinity to this receptor. The role of this receptor in epilepsy is still unclear [129], and a large number of studies consider TRPV1 activation to be detrimental; thus, CBD’s ability to activate it would be in direct contrast to its antiepileptic effect [121,130].

### 4.2. CBD in Parkinson’s Disease

CBD has also demonstrated significant effects in preclinical models of PD. This neurodegenerative condition initially affects the motor system, leading to tremor, bradykinesia (slowness of movement), and gait abnormalities (difficulty walking), as well as dementia and/or depression in later stages of the disease [131]. As previously mentioned, CBD is a partial agonist of the 5-HT_1A_ receptors, and some authors have described its activation as ameliorating non-motor symptoms of PD, including psychosis, depression, anxiety, and sleep disorders [22,24,132,133]. Additionally, CBD’s antipsychotic effect also seems to be associated with its inhibition of FAAH, leading to an increase in AEA levels [47,128]. This elevation in AEA levels is also hypothesized to contribute to hippocampal neurogenesis, consequently exerting antidepressive and anxiolytic effects [134,135].

Regarding motor symptoms, CBD has been proposed as anticataleptic through the activation of 5-HT_1A_, although the mechanisms responsible for its 5-HT_1A_ agonist-induced anticataleptic activity have yet to be fully understood [132,134]. Likewise, Peres, et al. [133] reported that CBD seems to prevent the increased catalepsy behavior induced by the repeated administration in reserpine on mice. Additionally, Gomes, et al. [136] suggested that CBD hinders cataleptic behavior in a dose-dependent manner, with the activation of 5-HT_1A_ being a contributing factor to this effect, as pre-treatment with 5-HT_1A_ antagonists blocked CBD’s action. The receptors GPR3 and GPR6 are also regarded as potential therapeutic targets, since they are associated with AD and PD, respectively [137]. A PD mouse model showed how a GPR6 knockout decreases cAMP production, leading to enhanced motor activity and decreased abnormal movements [137]. Regarding CBD, Laun, Shrader, Brown and Song [50] demonstrated that it was the only phytocannabinoid tested out of five (the remaining being Δ^9^-THC, cannabinol (CBN), cannabigerol (CBG), and cannabichromene (CBC)) that showed a significant effect on β-arrestin2 recruitment to both GPR3 and GPR6, exerting a concentration-dependent effect. Laun and Song [57] also described similar results; thus, the fact that CBD may act as an inverse agonist to these receptors may provide a potential explanation for its therapeutic effect on PD.

### 4.3. CBD in Alzheimer’s Disease

AD is a condition associated with an excessive increase in β-amyloid and hyperphosphorylated forms of the microtubule-associated protein tau [138]. GPR3 seems to contribute to the production of β-amyloid proteins, and the consequent deposition thereof. Hence, since CBD may act as an agonist to this receptor, it could exert a potential therapeutic effect [50,57,58].

Interestingly, CBD has been demonstrated to inhibit β-amyloid-induced tau hyperphosphorylation by glycogen synthase kinase-3β, potentially due to its partial activation of the PPAR receptors [111,139]. Additionally, Sativex^®^ (GW Pharmaceuticals, Cambridge, United Kingdom), a mixture of CBD and THC, was evaluated in a mouse model of an AD-related disorder, decreasing gliosis and iNOS levels and reducing the deposition of tau and β-amyloid proteins [140]. CBD’s anti-inflammatory activity has also been described in models of AD’s related neuroinflammation, with reports showing how CBD reduced microglial activation in mice, potentially decelerating the development of AD [141,142,143]. Microglial activation is characteristic of brain pathology, and β-amyloid-induced microglial activation is common in AD. CBD was able to reduce this activation in vitro and in vivo, while decreasing LPS-induced nitrite generation [141,142]. Cannabinoids, and particularly the CB_2_ receptor, have been shown to regulate microglial cell migration, potentially contributing to ameliorating neuroinflammation [85,144,145,146]. CBD has also been reported to control calcium flux through a mitochondrial voltage-dependent anion-selective channel 1 (VDAC1) related mechanism, since it is able to modulate this channel through conductance inhibition [65]. The reduced expression of this channel has been described as protecting against AD [147]. Restoring calcium homeostasis could be beneficial in other neurologic conditions; however, further studies of CBD’s role in this mechanism are still warranted [66,148,149].

### 4.4. CBD in Depression

There are plenty of reports associating CBD’s antidepressive and anxiolytic effects with its involvement with serotonergic neurotransmission. Xu, et al. [150] demonstrated how periodical administrations (orally and through intravenous injection) of CBD to a chronic mild stress mouse model resulted in antidepressant-like behavior in the forced swimming test. In line with these results, Shbiro, et al. [151] reported how different genetic rat models of depression also swam faster during the forced swimming test when CBD was administered. Sartim, Guimarães and Joca [67] also evaluated how CBD’s administration into rats’ ventral medial prefrontal cortex induced stress-coping behavior during the same test. However, this effect was blocked by pretreatment with the 5-HT_1A_ receptor antagonist WAY100635, which is in line with other reports where the use of a 5-HT_1A_ antagonist counteracted CBD’s anti-stress and anti-depressive effects [87,152,153].

### 4.5. CBD use in Neurological Conditions: Clinical Trials

Although there is increasing evidence of CBD’s beneficial effect against epilepsy, PD, and other neurological disorders, more studies and large-scale clinical trials are still needed to understand its long-term efficacy and assess its safety. Searching for ‘cannabidiol’ and ‘neurologic’ in the clinicaltrials.gov database (November 2022) results in 176 studies, with only 4 studies for PD. Of these, two of them focus on using CBD to treat motor symptoms (ClinicalTrials.gov identifiers: NCT02818777 and NCT03582137), one uses cannabis oil instead of CBD to treat pain (ClinicalTrials.gov identifier: NCT03639064), and one uses CBD to ameliorate non-motor symptoms of PD (ClinicalTrials.gov Identifier: NCT05106504). Epilepsy comprises 54 studies, with most of them focusing on the use of CBD to treat seizures, whereas two studies focus on the use of CBD to improve behavioral symptoms in AD (ClinicalTrials.gov Identifier: NCT04075435 and NCT04436081). Regarding the use of CBD to ameliorate depression symptoms, there is an ongoing trial in phase 2 (ClinicalTrials.gov Identifier: NCT05066308), while two trials focus on the use of CBD for anxiety and depression in bipolar disorder (ClinicalTrials.gov identifiers: NCT03310593 and NCT05457465). Moreover, six studies were found which use CBD to treat Post-Traumatic Stress Disorder (PTSD). Information regarding these clinical studies is presented in Table 2.

## 5. CBD in Autoimmune Diseases

A great body of evidence has been exposing the endocannabinoid system as a potential target for the treatment of inflammatory and autoimmune conditions related to immune cell activation. Indeed, medical cannabis and some of its derivatives, such as CBD and THC, have been demonstrating a capacity to downregulate the immune response through the activation of different receptors, resulting in a reduction in the mobilization and migration of leukocytes to sites of inflammation, and inhibition of pro-inflammatory cytokines release [153,157]. This therapeutic potential may be relevant for the treatment of various autoimmune diseases, including Crohn’s disease, rheumatoid arthritis, and psoriasis, among several other conditions. However, human trials are scarce and not conclusive, as shown below [157]. Multiple sclerosis (MS) will not be addressed, as CBD’s mechanisms of action in this condition have been vastly described elsewhere [158,159,160,161].

### 5.1. CBD in Inflammatory Bowel Disease

Inflammatory bowel disease (IBD) is a chronic, idiopathic, and relapsing inflammation of the gastrointestinal tract with characteristics of severe diarrhea, electrolyte loss, abnormal visceral sensitivity (hypersensitivity), bleeding and abdominal pain, and dysbiosis, and causes significant morbidity [162]. IBD can be classified into Crohn’s disease (CD) and Ulcerative Colitis (UC) based on characteristic clinical, radiological, endoscopic, and histological features. Although incompletely understood, the aetiology is thought to represent a complex interaction between genetic background, intestinal microbiota, environmental factors, and host immune response resulting in persistent and dysregulated inflammation [163]. The endocannabinoid system (ECS) has been recognized to play an important role in the maintenance of GI tract homeostasis, since it quickly responds to disturbances by de novo synthesis of its effector molecules and is, therefore, of particular interest in the management of IBD [164]. CB_1_ is predominantly present in enteric cholinergic neurons, where it inhibits neuronal hyperactivity, thus reducing GI motility and secretion [165]. CB_1_ receptors are also present in epithelial and plasma cells of the mucosa, where they are most likely involved in the regulation of mucosal permeability and wound healing [164,165,166]. CB_2_ receptors are mainly found in plasma cells as well as in macrophages in the mucosa [166]. One of the main purposes of the ECS in the gut is the maintenance of immune tolerance, in which CB_2_ plays an active role. Likewise, TRPV1 receptor is present in immune cells adjacent to blood vessels, as well as in the extrinsic afferent fibers, running through the muscle layers. Under inflammatory conditions, it increases intestinal contractility and visceral hypersensitivity signaling [166,167]. Other ECS components are also distributed along the GI tract, including PPAR-α (enterocytes of the small intestine and enteric glial cells), GPR55 (Epithelial cells and ENS of the small intestine), and FAAH (cells of the myenteric plexus in intestine) [166]. Nevertheless, the receptor sites of CBD in mammalian GI tract are not clear; multiple studies suggest different sites of action, including CB1, CB2, TRPV1, and PPAR-α [70,168,169,170].

Preclinical evidence for a beneficial effect of CBD in IBD has been obtained using animal models that rely on chemically-induced mucosal inflammation. An analysis of expression levels of ECS components in inflamed rodent colonic tissue revealed enhanced cannabinoid signaling under inflammatory conditions as compared to healthy tissue. Both CB1 and CB2 receptors, as well as AEA, were found to be upregulated in experimental IBD models [164]. Overall, studies have revealed that local or systemic administration of CBD results in a dose-related attenuation of disease parameters, including histopathological alterations, hypermotility, oxidative stress, and inflammatory signs, such as cytokines production (TNF-α, Il-6 or Il-1β) or the activation of macrophages, mastocytes, and enteric glial cells [77,166,170,171,172,173]. Ex vivo assays using IBD patients’ intestinal mucosal biopsies also demonstrated CBD’s capacity to prevent the production of inflammatory cytokines (TNF-α or INF-ϒ), cycloxygenase-2, and metalloproteinase-9, as well as to reduce the production of reactive oxygen species [172,174,175,176].

Despite the vast preclinical data showing beneficial effects of CBD in mouse and rat models of intestinal inflammation, clinical data are only beginning to emerge (Table 3). In a clinical trial on CBD in moderately active Crohn’s disease (NCT01037322), CBD was safe; however, it had no beneficial effects. The authors suggested that the lack of effect could be due to the reduced dose of CBD used (10 mg taken orally, twice a day), the small number of patients in the study (*n* = 19), or the lack of the necessary synergism with other cannabinoids [177]. A more recent trial (NCT01826188), assessing the effects of a CBD-rich cannabis oral treatment (cannabis oil containing 160/40 mg/mL of CBD/THC) upon 56 Crohn’s disease patients, concluded that treatment induced a significant clinical and quality of life improvement, yet without significant changes in inflammatory parameters or endoscopic scores [178]. A small proof of concept study (NCT01562314) using a CBD-rich botanical extract in patients with UC (starting with 50 mg of CBD extract in gelatine capsules, twice a day; dose increased to 250 mg, twice a day) revealed that, after per-protocol analysis, the primary endpoint of remission after treatment for a period of 10 weeks was not reached in patients in the CBD group [179]. Both reports concluded that further studies with a larger number of patients, different doses and routes of administration, and a follow-up to assess the long-term safety outcomes are still necessary. A new study sponsored by the Samuel Lunenfeld Research Institute on the use of Nabilone for acute pain in inflammatory bowel disease patients is estimated to start in the beginning of 2022 (NCT03422861). Nabilone (Cesamet^®^, Bausch Health, Quebec, Canada) is a synthetic cannabinoid that is chemically similar to Δ-9-THC and is capable of interacting with both CB1 and CB2 [174]. More recently, an observational study sponsored by Tel-Aviv Sourasky Medical Center (NCT05578313) has started, involving 1000 participants and aiming to understand or determine whether inhaled cannabis can induce remission in patients with IBD. Finally, a major phase 2 multistate, multicenter clinical study to determine the efficacy and safety of medical cannabis for a wide variety of chronic medical conditions, which includes CD and UC, is currently ongoing, and it is estimated to end in December 2025 (NCT03944447). This interventional study was estimated to enroll 200,000 participants and is sponsored by OMNI Medical Services, LLC.

### 5.2. CBD in Lupus

Lupus is a chronic (long-term) autoimmune disorder in which the body’s immune system becomes hyperactive and attacks healthy cells and tissues throughout the body, causing inflammation in different parts of the body [182]. Symptoms normally include severe fatigue, swelling, and joint pain, yet it may also cause damage to the skin, kidneys, heart, brain, bones, blood vessels, and lungs. The medical community does not fully understand what causes lupus; however, it is widely accepted that genetics play a key role and that women are at higher risk for lupus [182].

There are four main types of lupus, each with slightly different triggers and symptoms—systemic lupus erythematosus (SLE), cutaneous lupus, drug-induced lupus, and neonatal lupus. SLE is the most common form of lupus, where genetic, environmental, hormonal, epigenetic, and immunoregulatory factors act either sequentially or simultaneously on the immune system. The action of pathogenic factors results in the generation of autoantibodies directed against nuclear and cytoplasmic antigens, immune complexes, autoreactive or inflammatory T cells, and inflammatory cytokines which may affect several different organs, with a plethora of different clinical and immunologic abnormalities characterized by a relapsing and remitting clinical course [175,176,182].

Although various studies have investigated the role of ECS in different rheumatological and autoimmune diseases, there is not much information on potential alterations in SLE. Navarini and colleagues [183] evaluated the levels of plasma endocannabinoids in SLE patients compared to healthy volunteers. Plasma levels of 2-AG were found to be significantly elevated in SLE patients, while no statistical difference was observed in levels of AEA. Interestingly, 2-AG plasma levels were significantly increased in SLE patients with a lower clinical score compared to patients with a high clinical score. Moreover, examination of peripheral mononuclear blood cells in SLE patients revealed a positive correlation between CB2 mRNA expression and C3 and C4 plasma levels. Although the authors were not able to clarify the cause–effect relationship of the 2-AG increase in SLE patients, results suggest that pharmacological modulation of 2-AG levels may be beneficial to reducing disease activity. In the same vein, Rahaman and co-workers [184] disclosed a 2-AG mediated rheostat mechanism that selectively regulates IFN-α induction in plasmacytoid dendritic cells (pDC) via CB2 receptor. The disruption of such a rheostat mechanism by α/β-hydrolase domain-containing 6 overexpression leads to a reduction in 2-AG levels, causing aberrant IFN-α production by pDC in SLE patients. Two additional CB2-selective agonists, JWH-015 and JWH-133, showed comparable efficacy to THC in modulating IFNα and TNFα responses by pDC, demonstrating the potential for CB2-targeted therapeutics for the treatment of inflammatory conditions involving aberrant pDC activity, such as SLE [185]. THC and both JWH compounds inhibited CpG-induced IFNα and TNFα responses by pDC, and further suppressed phosphorylation of IRF7, TBK1, NFκB, and IKKγ, which are key events in pDC activation. However, information on the involvement of the endocannabinoid system in the pathogenesis of SLE is still scarce, a clinical trial phase II (NCT03093402) sponsored by Corbus Pharmaceuticals Inc and by the National Institute of Allergy and Infectious Diseases to evaluate the efficacy, safety, and tolerability of Lenabasum in systemic lupus erythematosus is ongoing. Lenabasum (or JBT-101) is a first-in-class, synthetic, orally active, and cannabinoid-derived drug that selectively activates the CB2 receptor [186].

### 5.3. CBD in Rheumatoid Arthritis

Rheumatoid arthritis (RA) is a chronic autoimmune disease that affects approximately 1% of the world population, being one of the main causes of disability worldwide. RA is characterized by persistent synovial inflammation, leading to progressive joint damage, and bone and cartilage destruction as well as deformity. Whilst the introduction of targeted biological drugs has led to a step change in the management of RA, 30–40% of patients do not respond adequately to these treatments, regardless of the mechanism of action of the drug used [187,188].

Although there is a lack of strong evidence on the efficacy of cannabinoids for the treatment of RA and other rheumatic diseases, cannabinoids have been seen as a potential therapy due to their anti-inflammatory effects. Indeed, RA is included in the list of conditions eligible for receiving medical cannabis in several countries and most patients with arthritis taking cannabinoids on a regularly basis report beneficial effects, such as less pain and an opioid-sparing effect [189]. In vitro and animal experimentation results have been the main source of data used so far to support cannabinoids potential therapeutic effect on RA. Gui and co-workers demonstrated the expression of CB2 receptor in synovial tissue from patients with RA, and its specific activation revealed inhibitory effects on fibroblast-like synoviocytes [190]. The same team demonstrated that the activation of CB2 in collagen-induced arthritis in mice, using HU-308 (a CBD-derivative drug that acts as a potent cannabinoid agonist) has therapeutic potential for RA to suppress synovitis and alleviate joint destruction by inhibiting the production of autoantibodies and proinflammatory cytokines [191]. Lowing and colleagues have recently proposed that a CB1 receptor antagonist combined with CBD and a FAAH inhibitor to inhibit the degradation of endocannabinoids could be beneficial in the treatment of RA [189]. The same group also demonstrated that CBD possesses anti-arthritic activity and might ameliorate arthritis via targeting synovial fibroblasts under inflammatory conditions, as CBD increases intracellular calcium levels, and reduces cell viability and IL-6/IL-8/MMP-3 production of rheumatoid arthritis synovial fibroblasts [192]. In 2005, the first ever controlled trial of a cannabis-based medicine (Sativex—THC plus CBD oral spray) in RA showcased potentially promising results, with a mild analgesic effect being observed. Moreover, disease activity seemed to be slightly suppressed in some patients following a five-week treatment. Whilst the differences were small and variable across the population, the results represented benefits of clinical relevance and showed the need for more detailed investigation [178]. Following this direction, at least three clinical trials assessing the efficacy of either cannabis or cannabinoids in patients with RA are currently recruiting or planning to start recruiting shortly: (i) Phase II NCT04269993—impact of cannabis on pain and inflammation among patients with rheumatoid or psoriatic arthritis, sponsored by Brown University; (ii) Phase I & II NCT04911127—therapeutic response of CBD in RA, sponsored by University of California; (iii) EudraCT 2017-004226-15—the efficacy and safety of using cannabis derivatives CBD and THC for the treatment of pain in patients with inflammatory arthritis, sponsored by King Christian 10^th^ Hospital for Rheumatology (Table 3).

### 5.4. CBD in Psoriasis

Psoriasis is a chronic, immune-mediated inflammatory disease characterized by well-demarcated scaly, erythematous, and infiltrated plaques, which affects approximately 2–3% of the worldwide population [193]. To date, the benchmark of psoriasis pathogenesis is yet to be discovered, since the current understanding relies on hypotheses relating to multifactorial etiologies, including genetic, epigenetic, and environmental factors. Nevertheless, psoriasis is characterized by effector T cell activation, dysregulated inflammatory cytokine expression—TNF-α, IL-17A/F, IL-22, IL-23, or GM-CFS—and angiogenic growth factors that flare up throughout the pathway, including hypoxia inducible factor-1α (HIF-1α) and vascular endothelial growth factor (VEGF) [194,195].

Phytocannabinoids have been described as promising agents in the treatment of psoriasis due to their capacity to inhibit keratinocytes hyperproliferation, while also ameliorating the associated inflammatory component [196]. Indeed, cannabinoid receptors are widely expressed throughout the skin epithelium, where CB1 activation has been associated with a decrease in the proliferation of epidermal keratinocytes. Skin exposed to synthetic CB1 agonist arachidonoyl-chloro-ethanolamide showed a reduction in keratinocytes proliferation, while decreasing the levels of psoriasis-associated proliferation markers, keratins 6 and 16 [197]. A different study has also demonstrated CBD, CBG, and THC’s capacity to inhibit keratinocytes hyperproliferation; yet, the mechanism responsible for this effect seems to be mostly independent from cannabinoid receptors. In fact, authors speculate on the involvement of the PPAR-γ, which has been suggested to be a cannabinoid receptor [198]. Dimethylbutyl-deoxy-Delta-8-THC, a synthetic cannabinoid holding potent anti-angiogenic and anti-inflammatory activities, has been suggested as potential treatment (oral or topical) for psoriasis due to its capacity to inhibit keratinocytes proliferation, as well as to target the two main pathways behind psoriasis pathogenesis, angiogenesis, and inflammation [199]. Recently, a method to treat psoriasis using a topical application containing CBD and CBG, at a concentration of 3–20%, has been patented by AXIM Biotechnologies. The results of an AXIM formulations study showed a 16–33% improvement in the lesions, suggesting a dose-response effect (ClinicalTrials.gov Identifier: NCT02976779). The safety and effectiveness of CBD in the treatment of psoriasis was also demonstrated in a three-month study, only involving five patients with severe/moderate psoriasis, where a CBD-enriched ointment improved hydration and elasticity, as well as significantly improved the Psoriasis Area Severity Index (PASI) score [200]. 

OWC Pharmaceutical Research sponsored a single center, prospective, double-blind, placebo-controlled, and randomized phase 1 study (NCT02976779) to assess the safety of topical cream designed to treat psoriasis (3% CBD and 3% THC) [201]. Although results were not shared, a patent was recently granted on pharmaceutical topical composition comprising CBD and THC for the treatment or prevention of inflammatory skin disorders [202]. No additional clinical studies are currently ongoing on CBD or other cannabinoids’ potential beneficial effects on the treatment of psoriasis vulgaris. Yet, two phase 2 studies focusing on psoriatic arthritis (a form of arthritis that affects some people who have psoriasis) are available, namely NCT03693833 sponsored by Aalborg University and NCT04269993 sponsored by Brown University (previously mentioned in the RA section). However, the goals of both trials are only centered on pain relief (Table 3).

## 6. CBD in Cancer Treatment: Beyond Pain Relief

The story of cannabinoid use in the treatment of several diseases such as cancer is old and long [203,204]. Cannabinoids were and are still used as pain relief measures in several illnesses [204]. In cancer, these drugs can help patients endure the pain and severe discomfort normally described as side effects of current chemotherapy treatments. The palliative use of cannabinoids and their derivatives prevents nausea, vomiting, and pain and stimulates the appetite [203]. THC and CBD are being considered to treat cachexia-anorexia syndrome [205]. This syndrome is common in patients with severe and advanced cancers, being characterized by a loss in appetite and weight, and declining physical condition [205].

The administration of cannabinoids can be via inhalation, orally as oils or oil-filled capsules, or via mucosal sprays containing either THC, CBD, or both [206]. There are some available medicinal products in the market, usually prescribed for other conditions such as Sativex^®^, which is used in multiple sclerosis [207]. Throughout the years, several studies and clinical trials have tried to establish a direct relationship between the use of THC and CBD in the pain relief of cancer patients. Despite the great potential of these compounds, there has been some difficulty in determining their pharmacokinetics parameters due to high variability among patients [205,208]. Nevertheless, although some adjustments must be made to make the use of cannabinoids more precise, their potency as pain relief is unequivocal and will be an important adjuvant in future medical care.

### 6.1. CBD Anticancer Mechanism

Since the 90s, it has been known that cannabinoids can induce cancer cell death [209]. It is well established that their interaction with CB1 and CB2 receptors triggers a signaling cascade that defines cell fate [210,211]. In the particular case of CBD, the interaction with CB_1_ and CB_2_ is known to be weak; however, CBD may also bind to other receptors that play a role in cellular fate and can be used as therapeutic targets for cancer treatment [210,211,212]. CBD can act as an inverse agonist of GPR3, GPR6, and GPR12 receptors, providing new and different mechanisms of action towards its therapeutic effect, not only in cancer but also in other conditions such as Alzheimer’s disease (AD), Parkinson’s disease (PD), and infertility [50]. Another study demonstrated that CBD interaction with GPR12 altered the viscoelasticity properties of cancer cells, leading to the blockade of metastasis (Brown, Laun, and Song 2017). CBD inhibits GRP55, decreasing the proliferation of pancreatic cancer cells and tumor growth in mice. This inhibition also resulted in increased effects of gemcitabine [81].

It has been shown that CBD can interact with transient receptor potential vanilloid 2 (TRPV2), a high-threshold thermosensor which plays a critical role in neuronal development, cardiac function, immunity, and cancer [53]. In colorectal cancer, CBD upregulated death receptor 5, enhancing the anticancer effect of TNF-related apoptosis-inducing ligand both in vitro and in vivo [213]. 

From the available data, it is possible to observe that, in cancer cells, CBD can interact with different receptors, triggering signaling pathways that lead to cell stress and ultimately to apoptosis and autophagy death processes. In a work using several *C. sativa* extracts, it was shown that CBD induced apoptosis in cervical cancer cells by overexpression of p53, caspase-3, and bax, as well as a decrease in ATP levels [214]. In addition, CBD inhibited the EGF/EGFR pathway, which resulted in the downregulation of EGFR, AKT, and NF-kB signaling pathways and the secretion of metalloproteinases MMP-2 and MMP-9. In several in vivo breast cancer models, this resulted in tumor growth and metastization inhibition [215]. In an in vitro model of colorectal cancer, it was observed that apoptosis resulting from CBD treatment was associated with the increased expression of pro-apoptotic protein NOXA, also stimulating the production of reactive oxygen species (ROS) [21]. Another mechanism for CBD-related apoptosis was described in gastric cancer, where CBD was shown to suppress X-linked inhibitor apoptosis proteins by decreasing their levels and increasing their ubiquitination. At the same time, it was also observed that CBD induced mitochondrial dysfunction [216]. This dysfunction, which leads to autophagic cell death, has been attributed to the CBD-induced Ca^+^ influx through TRPV4 [217].

Moreover, CBD can affect other cellular mechanisms which are related to cancer progression. Kosgodage and co-workers have demonstrated that CBD is capable of inhibiting the release of exosomes and microvesicles (EMV) in in vitro models of breast (MDA-MB-231), hepatic (HEPG2), and prostate (PC3) cancers [218]. EMV are lipid bilayer structures released by cancer cells as intercellular communication mechanisms that are involved in chemo-resistance processes. The authors of this study also showed that CBD was able to sensitize cancer cells to cisplatin-mediated apoptosis [218]. Also, CBD in combination with CBG and CBN (other non-psychoactive cannabinoids) induced cytoplasmatic vacuolization derived from the endoplasmic reticulum, leading to apoptosis, autophagy, and paraptosis, and presenting sensitivity to breast cancer cells in comparison to non-cancerous cell lines [219]. In lung cancer cells, CBD alone or in combination with THC affected the epithelial-mesenchymal transition and migration capacity, a phenotype that is commonly associated with the aggressiveness of cancers [220].

### 6.2. Combination of CBD with Chemotherapeutic Agents

Cancer is a complex and highly variable disease. Thus, the discovery of a single-molecule therapy to cure all cancers is a utopic idea. This gives strength to a multipurpose treatment using different drugs with different targets to achieve the same goal: cure cancer. The combination of CBD with other drugs and therapies has also been tested and discussed. In multiple myeloma cells, a combination of CBD and THC with carfilzomib, an immune-proteosome inhibitor, acted synergistically, leading to a decrease in cell viability and migration [221]. Jeong, et al. [222] demonstrated, in an in vitro colorectal cancer model, that CBD reduced the resistance to oxaliplatin by reducing the levels of NOS3 phosphorylation and nitric oxide production. In glioblastoma multiform (GBM), CBD was shown to interact synergistically with DNA-damaging agents, temozolomide, carmustine, and cisplatin [223], while a therapy combining cannabinoids and temozolomide was successfully tested for GBM [224]. The combination of THC and CBD, containing higher amounts of CBD but not CBD alone, together with temozolomide, led to a decrease in the growth of orthopic xenografts derived from glioma initiating cells, which have been associated with the relapses that occur in this type of cancer [224]. Fraguas-Sánchez, et al. [225] have created CBD-loaded PLGA-microparticles to circumvent the low aqueous solubility of the molecule. These microparticles were effective as sole therapy and especially in combination with doxorubicin or paclitaxel against the breast cancer cell lines and in ovo using MDA-MB-231-derived tumors [225].

### 6.3. The road to Clinic Application

Although CBD presents a surprising performance as an anticancer drug on the bench, the path to the bedside of cancer patients is still a winding path to go through. Thus far, there is not a complete description for the mechanistic action of CBD as an anticancer compound, although there is increasing evidence that CBD can trigger apoptotic events in different types of cancers. However, to achieve the status of approved efficacy, it needs to go through official clinical trials. In a search for the condition ‘cancer’ with ‘cannabidiol’ in the clinicaltrials.gov database (November 2022), 36 studies were found. From these, the majority are related to using CBD as a palliative drug to treat the harsh symptoms of advanced cancers. Nevertheless, there were eight entries dedicated to studying the efficacy of CBD as an anticancer treatment (Table 4): five for GBM (clinicaltrials.gov identifiers: NCT03529448; NCT01812616; NCT01812603; NCT03607643), one dedicated to advanced cancers in general (NCT02432612), one for prostate cancer (NCT04428203), one for breast cancer (NCT05016), and one for head and neck squamous cell carcinoma. In all these cases, CBD was evaluated in combination with THC in drugs such as Epidiolex or Sativex and combined with other approved chemotherapeutic agents, especially temozolomide. Also, few clinical studies have published results so far, with just two of them resulting in a publication regarding the safety and tolerability of Sativex in recurrent glioblastoma patients [226]. On a similar note, Bar-Sela, et al. [227] described the impact of cannabis consumption on patients with advanced-stage cancer and how it negatively correlates with immunotherapy and disease outcomes. There is still a long road to follow, and the possibility of chemical synthesis to obtain pure CBD can pave the way to further study the influence of this molecule in cancer therapy.

## 7. Final Remarks and Future Perspectives

Cannabis has been widely used as a palliative treatment to control pain. However, owing to THC, several concerns have arisen regarding its clinical and recurrent use, facing resistance at many levels (clinical, societal, and political). Research in this area has led to the discovery of other cannabinoids present in *C. sativa* that lack some of the psychoactive properties of THC and, concurrently, its secondary effects, besides possessing interesting properties from a pharmacological perspective not only for pain management but also as therapeutic effectors. One such cannabinoid, CBD, is commonly known for its anti-inflammatory, anticonvulsant, and anxiolytic properties. The molecular mechanisms by which CBD exerts its beneficial effects have revealed several pathways of action through which CBD interacts with multiple receptors. Nonetheless, a comprehensive perspective is still needed to deeply understand CBD activity.

Due to its potential, the clinical use of CBD has been studied in the context of diseases with an inflammatory profile such as cancer or autoimmune disorders. Additionally, neurological conditions are interesting targets for CBD properties. Several clinical studies have been and are still being performed to understand the safety and applicability of CBD in such conditions. Thus far, the available data suggest that CBD is well tolerated, with the main adverse effect being diarrhea [228]. However, CBD’s interaction with other medications still requires further enlightenment. The full clinical application of CBD and other cannabinoids is yet to be stablished; however, its therapeutic potential and use as an alternative to more classical treatments is unavoidable.

## Figures and Tables

**Table 1:** CBD interaction with receptors and targets and their outcomes.

Receptor/Target	Action	Effect	Dose	Reference
CB_1_	Inverse agonist/negative allosteric modulator	Antidepressant-like effect.	10–60 nmol (in vivo); 0.01–5 µM	[67,68]
CB_2_	Inverse agonist/negative allosteric modulator	Anti-inflammatory effect.	<1 µM	[25,69]
TRPV1-4	Agonist	Nociceptor desensitization effect.	1–10 µM	[70,71]
TRPM8	Antagonist	Inhibition of [Ca^2+^] elevation induced by menthol and icilin.	<0.1 µM	[71,72]
TRPA1	Agonist	Regulation of TRPV1 function.	EC50 12 µM	[72]
5-HT_1a_ receptors	Activation through direct, allosteric, or indirect effects	Associated with an antidepressant and anxiolytic effect.	50 mg/Kg (in vivo)	[73,74,75]
GPR55	Antagonist	Antagonization of proinflammatory effects.	10 mg/Kg (in vivo)	[76,77]
GPR3, GPR6 and GPR12	Inverse agonist	GPR3 is suggested as a biomarker for the prognosis of multiple sclerosis. GPR6 has been implicated in both HD and PD. GPR12 has been implicated in cell survival and neurite outgrow.	0.1–10 µM	[50,57]
PPAR-γ	Agonist/Up-regulator	Associated with anti-inflammatory and antioxidant properties through interaction with different transcription factors.	10 µM	[60,63,78]
TNF-α, IFN-β, IFN-γ, IL-1β, IL-17, IL-6	Modulator, decreases levels	Decrease in inflammation levels by targeting different pathways’ activity.	<20 µM	[79,80,81]
IL-4 and IL-10	Increases levels	Anti-inflammatory cytokines.	5 mg/Kg (in vivo)	[82,83]
ROS	Inhibitor	CBD inhibits a mechanism related to NADPH oxidase-mediated ROS production and NF-κB-dependent signaling events	<10 µM	[84,85]
iNOS and COX2	Inhibition of expression	Inhibition of the transcription of pro-inflammatory genes (i.e., iNOS, COX-2) contributing to CBD anti-inflammatory effect.	100 µM	[86,87]
Mitochondrial complexes I-IV	Inhibition	Decreases the activity of mitochondrial complexes (I, II, II-III, and IV).	50 µM	[88]
CaV3	Antagonist	Inhibition of the CaV3 channels might be involved in CBD analgesic effect.	1 µM	[89]
NaV	Inhibitor	Linked to antiseizure effects.	10 µM	[90]
VDAC1	Modulator	Associated to anticancer and immunosuppressive properties.	10 µM	[65]
AEA	Inhibitor	CBD acts in part by interfering with AEA inactivation and enhancing its inhibitory action on inflammation.	60 mg/Kg (in vivo)	[91,92]
FAAH	Inhibitor	Linked to CBD’s antipsychotic effect.	IC50 15 µM	[2,71]

Abbreviations: CB_1_—cannabionoid receptor 1; CB_2_—cannabinoid receptor 2; TRPV1-4—transient receptor potential cation channel subfamily V 1-4; TRPM8—transient receptor potential melastatin 8; TRPA1—Transient receptor potential cation channel subfamily A 1; 5-hydroxytryptamine (serotonin) receptor; EC50—half maximal effective concentration; GPR55—G protein-coupled receptor 55; GPR3—G protein-coupled receptor 3; GPR6—G protein-coupled receptor 6; GPR12—G protein-coupled receptor 12; HD—Huntington disease; PD—Parkinson disease; PPAR-γ—peroxisome proliferator-activated receptors gamma; TNF-α—tumor necrosis factor alpha; IFN-β—interferon beta; IFN-γ—interferon gamma; IL-1β—interleukin 1 beta; IL-17—interleukin 17; IL-6—interleukin 6; IL-4—interleukin 4; IL-10—interleukin 10; ROS—reactive oxygen species; CBD—cannabidiol; NADPH—Nicotinamide-adenine dinucleotide phosphate; iNOS—inducible nitric oxide synthase; COX-2—Cyclooxygenase 2; CaV3—T-type calcium channel 3; NaV—Voltage-gated sodium channel; VDAC1—voltage-dependent anion channel 1; AEA—anandamide; FAAH—fatty acid amide hydrolase; IC50—half maximal inhibitory concentration.

**Table 2:** List of clinical trials available at clinicaltrials.gov for the use of CBD in neurologic conditions.

Compound	Dosage and Treatment	Condition	Phase	Status and Results	Clinical Trial Code
High CBD and low THC	Hemp derived solution to be administered sublingually twice daily	AD and anxiety	Early 1	Still recruiting	NCT04075435
THC-free CBD oil	Starting at a dosage of 15 mg twice per day with up titration to 45 mg twice per day; oral solution	AD and dementia	2	Still recruiting	NCT04436081
GWP42003 (purified CBD)	Started at 5 mg/kg/day and is increased by 2.5–5 mg/kg at 3–5-day intervals to a target dose of 20 mg/kg/day; oral solution	Tremor in PD	2	3 participants dropped out due to study drug intolerance. The remaining participants demonstrated improvements in cognition, depression, and emotional issues associated with PD.	NCT02818777
Medical cannabis	Inhaled dried buds or sublingual oil extract	Non-motor symptoms of PD	2	Still recruiting	NCT05106504
Nabilone	0.25–2 mg per day. Capsules, taken orally daily	Non-motor symptoms of PD	2	Positive effects on anxious mood and night-time sleep problems, with no serious adverse effects registered [154].	NCT03769896
GWP42003 (purified CBD)	150 mg/day, increased by 150 mg/day each week for the remaining three weeks. Maximum dose of 600 mg/day during the fourth week; oral solution	Psychosis in PD	2	No adverse effect was observed during the treatment. Psychosis symptoms decreased [148].	NCT02818777
Epidiolex	100 mg/mL oral solution titrated to a target maintenance dose of 20 mg/kg/day over 2 weeks	Epilepsy	3	The study demonstrated long-term benefits with a 43.9% reduction in monthly drop and total seizures frequency in the CBD group, observed through 156 weeks [149].	NCT02224573
CBD (nonplant-based)	3 multiple ascending doses: 10 mg/kg/day, 20 mg/kg/day and 40 mg/kg/day; oral solution	Epilepsy	1/2	Short-term administration was generally safe and well tolerated. Inter-individual variability decreased with multiple doses [155].	NCT02324673
Epidiolex	Various doses between 5 mg/kg/day and 50 mg/kg/day; oral solution	Epilepsy	-	Seizure control is proportional to CBD plasma level, with a linear relationship between dosage and level of CBD [156].	NCT02695537
CBD (in corn oil)	5–25 mg/kg/day; oral solution	Epilepsy	3	On-going	NCT02783092
CBD (supplement with a bio-terpene complex)	1 capsule/day (Hemp Extract 35 mg; Bio-Terpene Complex 52 mg)	Anxiety and stress	-	Not shared	NCT05518019
Cannabis oil	Single dose of CBD (300 mg)	Social anxiety disorder	-	CBD significantly decreased anxiety measured by two different scales [152].	JCT0018004564

**Table 3:** List of clinical trials available for the use of CBD in autoimmune diseases.

Compound	Dosage and Treatment	Condition	Phase	Status and Results	Clinical Trial Code
CBD	10 mg daily. Sublingual	IBD (CD and UC)	1/2	Safe but not effective as treatment [177].	NCT01037322
CBD and THC oil	Initially 16 mg CBD and 4 mg THC/day. Maximum: 320 mg CBD and 80 mg THC/day. Sublingual	CD	1/2	Significant clinical and QOL improvement without significant changes in inflammatory parameters or endoscopic scores [178].	NCT01826188
CBD (rich botanical extract)	50 mg up to 250 mg daily. Ingestion	UC	2	No remission after treatment, yet promoted QOL improvement [179].	NCT01562314
Medical cannabis	Inhalation	IBD (CD and UC)	2	On-going	NCT05578313
Medical cannabis	Inhalation	IBD (CD and UC)	2	On-going	NCT03944447
Sativex (CBD and THC)	2.7 mg THC and 2.5 mg CBD. Oromucosal spray	RA	2	A significant analgesic effect was observed, and disease activity was significantly suppressed following Sativex treatment [180].	-
Medical cannabis	Inhalation	RA and PSA	2	On-going.	NCT04269993
CBD	400 or 800 mg daily. Ingestion	RA	1/2	On-going.	NCT04911127
CBD	Initial: 10 mg CBD/day. Maximum: 30 mg CBD/day. Ingestion	RA	2	On-going [181].	EudraCT 2017-004226-15
CBD and THC	3% CBD + 3% THC; topical	Psoriasis	1	Not shared.	NCT02976779
CBD	Initial: 10 mg CBD/day. Maximum: 30 mg CBD/day. Ingestion	PSA	2	Not shared.	NCT03693833

**Table 4:** List of clinical trials available at clinicaltrials.gov for the use of CBD as a cancer therapeutic approach.

Compound	Other Drugs/Treatment	Type of Cancer	Phase	Status and Results	Clinical Trial Code
Epidiolex	-	Prostate cancer	1	Recruitment completed No results shared	NCT04428203
Epidiolex	Mifepristone (antiprogesterone)	Breast cancer	3	Not yet recruiting	NCT05016349
	Tamoxifen Retinoic acid	data			
BRCX014 (CBD 200 mg)	Bortezomib Leucovorin 5-FU Oxaliplatin Bevacizumab Irinotecan Gemcitabine Temozolomide	Multiple myeloma Glioblastoma multiforme GI malignancies	1/2	Unknown	NCT03607643
Sativex (27 mg/mL THC and 25 mg/mL CBD)		Advanced cancer	1	Withdrawn	NCT02432612
Sativex	Temozolomide	Recurrent Glioblastoma	1/2	Completed [226]	NCT01812603 NCT01812616
Sativex		Head and neck squamous cell carcinoma	1	Terminated (slow recruitment)	NCT01975688
TN-TC11G (5 mg of CBD, 5 mg of THC)	Temozolomide Radiation therapy	Newly diagnosed Glioblastoma	1/2	Estimated completion in 2024	NCT03529448

## Data Availability

Data sharing not applicable.

## References

[1] Atalay S., Jarocka-Karpowicz I., Skrzydlewska E. (2020). Antioxidative and Anti-Inflammatory Properties of Cannabidiol. Antioxidants.

[2] Nichols J.M., Kaplan B.L. (2020). Immune responses regulated by cannabidiol. Cannabis Cannabinoid Res..

[3] Silote G.P., Sartim A., Sales A., Eskelund A., Guimarães F.S., Wegener G., Joca S. (2019). Emerging evidence for the antidepressant effect of cannabidiol and the underlying molecular mechanisms. J. Chem. Neuroanat..

[4] Franco V., Perucca E. (2019). Pharmacological and Therapeutic Properties of Cannabidiol for Epilepsy. Drugs.

[5] Mandolini G., Lazzaretti M., Pigoni A., Oldani L., Delvecchio G., Brambilla P. (2018). Pharmacological properties of cannabidiol in the treatment of psychiatric disorders: A critical overview. Epidemiol. Psychiatr. Sci..

[6] Grotenhermen F. (2003). Pharmacokinetics and pharmacodynamics of cannabinoids. Clin. Pharmacokinet..

[7] Fraguas-Sánchez A., Fernández-Carballido A., Sofware C.M.-S., Torres-Suárez A. (2020). Stability characteristics of Cannabidiol for the design of pharmacological, biochemical and pharmaceutical studies. J. Chromatogr. B.

[8] Pavlovic R., Nenna G., Calvi L., Panseri S., Borgonovo G., Giupponi L., Cannazza G., Giorgi A. (2018). Quality traits of “cannabidiol oils”: Cannabinoids content, terpene fingerprint and oxidation stability of European commercially available preparations. Molecules.

[9] Scheidweiler K.B., Andersson M., Swortwood M.J., Sempio C., Huestis M.A. (2017). Long-term stability of cannabinoids in oral fluid after controlled cannabis administration. Drug Test. Anal..

[10] Bonn-Miller M.O., ElSohly M.A., Loflin M.J., Chandra S., Vandrey R. (2018). Cannabis and cannabinoid drug development: Evaluating botanical versus single molecule approaches. Int. Rev. Psychiatry.

[11] Mascal M., Hafezi N., Wang D., Hu Y., Serra G., Dallas M.L., Spencer J.P. (2019). Synthetic, non-intoxicating 8, 9-dihydrocannabidiol for the mitigation of seizures. Sci. Rep..

[12] Amendola G., Bocca B., Picardo V., Pelosi P., Battistini B., Ruggieri F., Barbini D.A., De Vita D., Madia V., Messore A. (2021). Toxicological aspects of cannabinoid, pesticide and metal levels detected in light Cannabis inflorescences grown in Italy. Food Chem. Toxicol..

[13] Montoya Z., Conroy M., Vanden Heuvel B.D., Pauli C.S., Park S.-H. (2020). Cannabis contaminants limit pharmacological use of cannabidiol. Front. Pharmacol..

[14] Sgrò S., Lavezzi B., Caprari C., Polito M., D’Elia M., Lago G., Furlan G., Girotti S., Ferri E.N. (2021). Delta9-THC determination by the EU official method: Evaluation of measurement uncertainty and compliance assessment of hemp samples. Anal. Bioanal. Chem..

[15] Żuk-Gołaszewska K., Gołaszewski J. (2020). Hemp production. Sustain. Agric. Rev..

[16] Kennedy M.C. (2017). Cannabis: Exercise performance and sport. A systematic review. J. Sci. Med. Sport.

[17] van Wilgen C.P., Keizer D. (2011). Neuropathic pain mechanisms in patients with chronic sports injuries: A diagnostic model useful in sports medicine?. Pain Med..

[18] Jesus C.H.A., Redivo D.D.B., Gasparin A.T., Sotomaior B.B., de Carvalho M.C., Genaro K., Zuardi A.W., Hallak J.E.C., Crippa J.A., Zanoveli J.M. (2019). Cannabidiol attenuates mechanical allodynia in streptozotocin-induced diabetic rats via serotonergic system activation through 5-HT1A receptors. Brain Res..

[19] Costa B., Trovato A.E., Comelli F., Giagnoni G., Colleoni M. (2007). The non-psychoactive cannabis constituent cannabidiol is an orally effective therapeutic agent in rat chronic inflammatory and neuropathic pain. Eur. J. Pharmacol..

[20] Jung B., Lee J.K., Kim J., Kang E.K., Han S.Y., Lee H.Y., Choi I.S. (2019). Synthetic Strategies for (−)-Cannabidiol and Its Structural Analogs. Chem. –Asian J..

[21] Jeong S., Yun H.K., Jeong Y.A., Jo M.J., Kang S.H., Kim J.L., Kim D.Y., Park S.H., Kim B.R., Na Y.J. (2019). Cannabidiol-induced apoptosis is mediated by activation of Noxa in human colorectal cancer cells. Cancer Lett..

[22] Saleem S., Anwar A. (2020). Cannabidiol: A hope to treat non-motor symptoms of Parkinson′s disease patients. Eur. Arch. Psychiatry Clin. Neurosci..

[23] Hao E., Mukhopadhyay P., Cao Z., Erdélyi K., Holovac E., Liaudet L., Lee W.-S., Haskó G., Mechoulam R., Pacher P. (2015). Cannabidiol protects against doxorubicin-induced cardiomyopathy by modulating mitochondrial function and biogenesis. Mol. Med..

[24] Patricio F., Morales-Andrade A.A., Patricio-Martínez A., Limón I.D. (2020). Cannabidiol as a therapeutic target: Evidence of its neuroprotective and neuromodulatory function in Parkinson’s disease. Front. Pharmacol..

[25] Fernández-Ruiz J., Sagredo O., Pazos M.R., García C., Pertwee R., Mechoulam R., Martínez-Orgado J. (2013). Cannabidiol for neurodegenerative disorders: Important new clinical applications for this phytocannabinoid?. Br. J. Clin. Pharmacol..

[26] Kozela E., Juknat A., Gao F., Kaushansky N., Coppola G., Vogel Z. (2016). Pathways and gene networks mediating the regulatory effects of cannabidiol, a nonpsychoactive cannabinoid, in autoimmune T cells. J. Neuroinflamm..

[27] Jean-Gilles L., Braitch M., Latif M.L., Aram J., Fahey A.J., Edwards L.J., Robins R.A., Tanasescu R., Tighe P.J., Gran B. (2015). Effects of pro-inflammatory cytokines on cannabinoid CB 1 and CB 2 receptors in immune cells. Acta Physiol..

[28] Srivastava M.D., Srivastava B., Brouhard B. (1998). Δ9 tetrahydrocannabinol and cannabidiol alter cytokine production by human immune cells. Immunopharmacology.

[29] Lu H.-C., Mackie K. (2016). An introduction to the endogenous cannabinoid system. Biol. Psychiatry.

[30] McPartland J.M., Duncan M., Di Marzo V., Pertwee R.G. (2015). Are cannabidiol and Δ9-tetrahydrocannabivarin negative modulators of the endocannabinoid system? A systematic review. Br. J. Pharmacol..

[31] Bíró T., Tóth B.I., Haskó G., Paus R., Pacher P. (2009). The endocannabinoid system of the skin in health and disease: Novel perspectives and therapeutic opportunities. Trends Pharmacol. Sci..

[32] Pacher P., Bátkai S., Kunos G. (2006). The endocannabinoid system as an emerging target of pharmacotherapy. Pharmacol. Rev..

[33] Oddi S., Scipioni L., Maccarrone M. (2020). Endocannabinoid system and adult neurogenesis: A focused review. Curr. Opin. Pharmacol..

[34] van Eenige R., van der Stelt M., Rensen P.C., Kooijman S. (2018). Regulation of adipose tissue metabolism by the endocannabinoid system. Trends Endocrinol. Metab..

[35] Uhelski M.L., Khasabova I., Simone D.A., Costain W.J. (2018). Modulation of Pain by Endocannabinoids in the Periphery. Recent Advances in Cannabinoid Research.

[36] Sierra S., Luquin N., Navarro-Otano J. (2018). The endocannabinoid system in cardiovascular function: Novel insights and clinical implications. Clin. Auton. Res..

[37] Turcotte C., Blanchet M.-R., Laviolette M., Flamand N. (2016). The CB 2 receptor and its role as a regulator of inflammation. Cell. Mol. Life Sci..

[38] Karsak M., Gaffal E., Date R., Wang-Eckhardt L., Rehnelt J., Petrosino S., Starowicz K., Steuder R., Schlicker E., Cravatt B. (2007). Attenuation of allergic contact dermatitis through the endocannabinoid system. science.

[39] Watson S., Chambers D., Hobbs C., Doherty P., Graham A. (2008). The endocannabinoid receptor, CB1, is required for normal axonal growth and fasciculation. Mol. Cell. Neurosci..

[40] Mechoulam R., Hanuš L.O., Pertwee R., Howlett A.C. (2014). Early phytocannabinoid chemistry to endocannabinoids and beyond. Nat. Rev. Neurosci..

[41] Amenta P.S., Jallo J.I., Tuma R.F., Hooper D.C., Elliott M.B. (2014). Cannabinoid receptor type-2 stimulation, blockade, and deletion alter the vascular inflammatory responses to traumatic brain injury. J. Neuroinflamm..

[42] Mukhopadhyay P., Rajesh M., Horváth B., Bátkai S., Park O., Tanchian G., Gao R.Y., Patel V., Wink D.A., Liaudet L. (2011). Cannabidiol protects against hepatic ischemia/reperfusion injury by attenuating inflammatory signaling and response, oxidative/nitrative stress, and cell death. Free. Radic. Biol. Med..

[43] Hegde V.L., Hegde S., Cravatt B.F., Hofseth L.J., Nagarkatti M., Nagarkatti P.S. (2008). Attenuation of experimental autoimmune hepatitis by exogenous and endogenous cannabinoids: Involvement of regulatory T cells. Mol. Pharmacol..

[44] Di Marzo V. (2006). Endocannabinoids: Synthesis and degradation. Reviews of Physiology Biochemistry and Pharmacology.

[45] Mayo L.M., Asratian A., Lindé J., Morena M., Haataja R., Hammar V., Augier G., Hill M.N., Heilig M. (2020). Elevated anandamide, enhanced recall of fear extinction, and attenuated stress responses following inhibition of fatty acid amide hydrolase: A randomized, controlled experimental medicine trial. Biol. Psychiatry.

[46] Mayo L.M., Asratian A., Lindé J., Holm L., Nätt D., Augier G., Stensson N., Vecchiarelli H.A., Balsevich G., Aukema R.J. (2018). Protective effects of elevated anandamide on stress and fear-related behaviors: Translational evidence from humans and mice. Mol. Psychiatry.

[47] Habib A.M., Okorokov A.L., Hill M.N., Bras J.T., Lee M.-C., Li S., Gossage S.J., van Drimmelen M., Morena M., Houlden H. (2019). Microdeletion in a FAAH pseudogene identified in a patient with high anandamide concentrations and pain insensitivity. Br. J. Anaesth..

[48] Cravatt B.F., Demarest K., Patricelli M.P., Bracey M.H., Giang D.K., Martin B.R., Lichtman A.H. (2001). Supersensitivity to anandamide and enhanced endogenous cannabinoid signaling in mice lacking fatty acid amide hydrolase. Proc. Natl. Acad. Sci..

[49] Schlosburg J.E., Blankman J.L., Long J.Z., Nomura D.K., Pan B., Kinsey S.G., Nguyen P.T., Ramesh D., Booker L., Burston J.J. (2010). Chronic monoacylglycerol lipase blockade causes functional antagonism of the endocannabinoid system. Nat. Neurosci..

[50] Laun A.S., Shrader S.H., Brown K.J., Song Z.-H. (2019). GPR3, GPR6, and GPR12 as novel molecular targets: Their biological functions and interaction with cannabidiol. Acta Pharmacol. Sin..

[51] Brown K.J., Laun A.S., Song Z.-H. (2017). Cannabidiol, a novel inverse agonist for GPR12. Biochem. Biophys. Res. Commun..

[52] Waldeck-Weiermair M., Zoratti C., Osibow K., Balenga N., Goessnitzer E., Waldhoer M., Malli R., Graier W.F. (2008). Integrin clustering enables anandamide-induced Ca2+ signaling in endothelial cells via GPR55 by protection against CB1-receptor-triggered repression. J. Cell Sci..

[53] Qin N., Neeper M.P., Liu Y., Hutchinson T.L., Lubin M.L., Flores C.M. (2008). TRPV2 is activated by cannabidiol and mediates CGRP release in cultured rat dorsal root ganglion neurons. J. Neurosci..

[54] Fogaça M.V., Campos A.C., Guimarães F.S. (2016). Cannabidiol and 5-HT1A receptors. Neuropathology of Drug Addictions and Substance Misuse.

[55] Pumroy R.A., Samanta A., Liu Y., Hughes T.E., Zhao S., Yudin Y., Rohacs T., Han S., Moiseenkova-Bell V.Y. (2019). Molecular mechanism of TRPV2 channel modulation by cannabidiol. Elife.

[56] Pelz M.C., Schoolcraft K.D., Larson C., Spring M.G., López H.H. (2017). Assessing the role of serotonergic receptors in cannabidiol′s anticonvulsant efficacy. Epilepsy Behav..

[57] Laun A.S., Song Z.-H. (2017). GPR3 and GPR6, novel molecular targets for cannabidiol. Biochem. Biophys. Res. Commun..

[58] Huang Y., Skwarek-Maruszewska A., Horré K., Vandewyer E., Wolfs L., Snellinx A., Saito T., Radaelli E., Corthout N., Colombelli J. (2015). Loss of GPR3 reduces the amyloid plaque burden and improves memory in Alzheimer’s disease mouse models. Sci. Transl. Med..

[59] Massi P., Solinas M., Cinquina V., Parolaro D. (2013). Cannabidiol as potential anticancer drug. Br. J. Clin. Pharmacol..

[60] O′Sullivan S.E. (2016). An update on PPAR activation by cannabinoids. Br. J. Pharmacol..

[61] Jurkus R., Day H.L., Guimarães F.S., Lee J.L., Bertoglio L.J., Stevenson C.W. (2016). Cannabidiol regulation of learned fear: Implications for treating anxiety-related disorders. Front. Pharmacol..

[62] Silva R.L., Silveira G.T., Wanderlei C.W., Cecilio N.T., Maganin A.G., Franchin M., Marques L.M., Lopes N.P., Crippa J.A., Guimarães F.S. (2019). DMH-CBD, a cannabidiol analog with reduced cytotoxicity, inhibits TNF production by targeting NF-kB activity dependent on A2A receptor. Toxicol. Appl. Pharmacol..

[63] Hind W.H., England T.J., O′Sullivan S.E. (2016). Cannabidiol protects an in vitro model of the blood–brain barrier from oxygen-glucose deprivation via PPARγ and 5-HT1A receptors. Br. J. Pharmacol..

[64] Nichol K., Stott C., Jones N., Gray R.A., Bazelot M., Whalley B.J. (2019). The proposed multimodal mechanism of action of cannabidiol (CBD) in epilepsy: Modulation of intracellular calcium and adenosine-mediated signaling (P5. 5-007). Neurology.

[65] Rimmerman N., Ben-Hail D., Porat Z., Juknat A., Kozela E., Daniels M.P., Connelly P.S., Leishman E., Bradshaw H.B., Shoshan-Barmatz V. (2013). Direct modulation of the outer mitochondrial membrane channel, voltage-dependent anion channel 1 (VDAC1) by cannabidiol: A novel mechanism for cannabinoid-induced cell death. Cell Death Dis..

[66] Ryan D., Drysdale A.J., Lafourcade C., Pertwee R.G., Platt B. (2009). Cannabidiol targets mitochondria to regulate intracellular Ca2+ levels. J. Neurosci..

[67] Sartim A.G., Guimarães F.S., Joca S.R.L. (2016). Antidepressant-like effect of cannabidiol injection into the ventral medial prefrontal cortex—Possible involvement of 5-HT1A and CB1 receptors. Behav. Brain Res..

[68] Laprairie R., Bagher A., Kelly M., Denovan-Wright E. (2015). Cannabidiol is a negative allosteric modulator of the cannabinoid CB1 receptor. Br. J. Pharmacol..

[69] Howlett A.C., Abood M.E. (2017). CB1 and CB2 receptor pharmacology. Advances in Pharmacology.

[70] De Petrocellis L., Orlando P., Moriello A.S., Aviello G., Stott C., Izzo A., Di Marzo V. (2012). Cannabinoid actions at TRPV channels: Effects on TRPV3 and TRPV4 and their potential relevance to gastrointestinal inflammation. Acta Physiol..

[71] De Petrocellis L., Ligresti A., Moriello A.S., Allarà M., Bisogno T., Petrosino S., Stott C.G., Di Marzo V. (2011). Effects of cannabinoids and cannabinoid-enriched Cannabis extracts on TRP channels and endocannabinoid metabolic enzymes. Br. J. Pharmacol..

[72] De Petrocellis L., Vellani V., Schiano-Moriello A., Marini P., Magherini P.C., Orlando P., Di Marzo V. (2008). Plant-derived cannabinoids modulate the activity of transient receptor potential channels of ankyrin type-1 and melastatin type-8. J. Pharmacol. Exp. Ther..

[73] Cascio M.G., Gauson L.A., Stevenson L.A., Ross R.A., Pertwee R.G. (2010). Evidence that the plant cannabinoid cannabigerol is a highly potent α2-adrenoceptor agonist and moderately potent 5HT1A receptor antagonist. Br. J. Pharmacol..

[74] Linge R., Jiménez-Sánchez L., Campa L., Pilar-Cuéllar F., Vidal R., Pazos A., Adell A., Díaz A. (2016). Cannabidiol enhancement of serotonergic and glutamatergic signaling in a mouse model of depression induces fast and maintained antidepressant actions: Implication of 5-HT1A receptors. https://digital.csic.es/bitstream/10261/164285/1/cannabidiolreceptor.pdf.

[75] Linge R., Jiménez-Sánchez L., Campa L., Pilar-Cuéllar F., Vidal R., Pazos A., Adell A., Díaz Á. (2016). Cannabidiol induces rapid-acting antidepressant-like effects and enhances cortical 5-HT/glutamate neurotransmission: Role of 5-HT1A receptors. Neuropharmacology.

[76] Chiurchiù V., Lanuti M., De Bardi M., Battistini L., Maccarrone M. (2015). The differential characterization of GPR55 receptor in human peripheral blood reveals a distinctive expression in monocytes and NK cells and a proinflammatory role in these innate cells. Int. Immunol..

[77] Lin X.H., Yuece B., Li Y.Y., Feng Y.J., Feng J.Y., Yu L.Y., Li K., Li Y.N., Storr M. (2011). A novel CB receptor GPR55 and its ligands are involved in regulation of gut movement in rodents. Neurogastroenterol. Motil..

[78] Sonego A.B., Prado D.S., Vale G.T., Sepulveda-Diaz J.E., Cunha T.M., Tirapelli C.R., Del Bel E.A., Raisman-Vozari R., Guimarães F.S. (2018). Cannabidiol prevents haloperidol-induced vacuos chewing movements and inflammatory changes in mice via PPARγ receptors. Brain Behav. Immun..

[79] Rajan T.S., Giacoppo S., Iori R., De Nicola G.R., Grassi G., Pollastro F., Bramanti P., Mazzon E. (2016). Anti-inflammatory and antioxidant effects of a combination of cannabidiol and moringin in LPS-stimulated macrophages. Fitoterapia.

[80] Petrosino S., Verde R., Vaia M., Allarà M., Iuvone T., Di Marzo V. (2018). Anti-inflammatory properties of cannabidiol, a nonpsychotropic cannabinoid, in experimental allergic contact dermatitis. J. Pharmacol. Exp. Ther..

[81] Pellati F., Borgonetti V., Brighenti V., Biagi M., Benvenuti S., Corsi L. (2018). Cannabis sativa L. and nonpsychoactive cannabinoids: Their chemistry and role against oxidative stress, inflammation, and cancer. BioMed Res. Int..

[82] Weiss L., Zeira M., Reich S., Har-Noy M., Mechoulam R., Slavin S., Gallily R. (2006). Cannabidiol lowers incidence of diabetes in non-obese diabetic mice. Autoimmunity.

[83] Sacerdote P., Martucci C., Vaccani A., Bariselli F., Panerai A., Colombo A., Parolaro D., Massi P. (2005). The nonpsychoactive component of marijuana cannabidiol modulates chemotaxis and IL-10 and IL-12 production of murine macrophages both in vivo and in vitro. J. Neuroimmunol..

[84] Hamelink C., Hampson A., Wink D.A., Eiden L.E., Eskay R.L. (2005). Comparison of cannabidiol, antioxidants, and diuretics in reversing binge ethanol-induced neurotoxicity. J. Pharmacol. Exp. Ther..

[85] dos-Santos-Pereira M., Guimarães F.S., Del-Bel E., Raisman-Vozari R., Michel P.P. (2020). Cannabidiol prevents LPS-induced microglial inflammation by inhibiting ROS/NF-κB-dependent signaling and glucose consumption. Glia.

[86] Jastrząb A., Gęgotek A., Skrzydlewska E. (2019). Cannabidiol regulates the expression of keratinocyte proteins involved in the inflammation process through transcriptional regulation. Cells.

[87] Castillo A., Tolón M., Fernández-Ruiz J., Romero J., Martinez-Orgado J. (2010). The neuroprotective effect of cannabidiol in an in vitro model of newborn hypoxic–ischemic brain damage in mice is mediated by CB2 and adenosine receptors. Neurobiol. Dis..

[88] Singh N., Hroudová J., Fišar Z. (2015). Cannabinoid-induced changes in the activity of electron transport chain complexes of brain mitochondria. J. Mol. Neurosci..

[89] Ross H.R., Napier I., Connor M. (2008). Inhibition of recombinant human T-type calcium channels by Δ9-tetrahydrocannabinol and cannabidiol. J. Biol. Chem..

[90] Hill A.J., Jones N.A., Smith I., Hill C.L., Williams C.M., Stephens G.J., Whalley B.J. (2014). Voltage-gated sodium (NaV) channel blockade by plant cannabinoids does not confer anticonvulsant effects per se. Neurosci. Lett..

[91] Pedrazzi J.F.C., Issy A., Gomes F., Guimarães F., Del-Bel E. (2015). Cannabidiol effects in the prepulse inhibition disruption induced by amphetamine. Psychopharmacology.

[92] Bisogno T. (2008). Endogenous cannabinoids: Structure and metabolism. J. Neuroendocrinol..

[93] Klotz K.A., Grob D., Hirsch M., Metternich B., Schulze-Bonhage A., Jacobs J. (2019). Efficacy and tolerance of synthetic cannabidiol for treatment of intractable epilepsy. Front. Neurol..

[94] Wilson J.T., Fief C.A., Jackson K.D., Mercer S.L., Deweese J.E. (2018). HU-331 and oxidized cannabidiol act as inhibitors of human topoisomerase IIα and β. Chem. Res. Toxicol..

[95] Sumariwalla P.F., Gallily R., Tchilibon S., Fride E., Mechoulam R., Feldmann M. (2004). A novel synthetic, nonpsychoactive cannabinoid acid (HU-320) with antiinflammatory properties in murine collagen-induced arthritis. Arthritis Rheum..

[96] Çakır M., Tekin S., Doğanyiğit Z., Erden Y., Soytürk M., Çiğremiş Y., Sandal S. (2019). Cannabinoid type 2 receptor agonist JWH-133, attenuates Okadaic acid induced spatial memory impairment and neurodegeneration in rats. Life Sci..

[97] Xu H., Cheng C.L., Chen M., Manivannan A., Cabay L., Pertwee R.G., Coutts A., Forrester J.V. (2007). Anti-inflammatory property of the cannabinoid receptor-2-selective agonist JWH-133 in a rodent model of autoimmune uveoretinitis. J. Leukoc. Biol..

[98] Aso E., Juvés S., Maldonado R., Ferrer I. (2013). CB 2 cannabinoid receptor agonist ameliorates Alzheimer-like phenotype in AβPP/PS1 mice. J. Alzheimer′s Dis..

[99] Qamri Z., Preet A., Nasser M.W., Bass C.E., Leone G., Barsky S.H., Ganju R.K. (2009). Synthetic cannabinoid receptor agonists inhibit tumor growth and metastasis of breast cancer. Mol. Cancer Ther..

[100] Bisogno T., Oddi S., Piccoli A., Fazio D., Maccarrone M. (2016). Type-2 cannabinoid receptors in neurodegeneration. Pharmacol. Res..

[101] Kruk-Slomka M., Banaszkiewicz I., Biala G. (2017). The impact of CB2 receptor ligands on the MK-801-induced hyperactivity in mice. Neurotox. Res..

[102] Bolognini D., Cascio M.G., Parolaro D., Pertwee R.G. (2012). AM630 behaves as a protean ligand at the human cannabinoid CB2 receptor. Br. J. Pharmacol..

[103] Ottani A., Giuliani D. (2001). HU 210: A potent tool for investigations of the cannabinoid system. CNS Drug Rev..

[104] Skrabek R.Q., Galimova L., Ethans K., Perry D. (2008). Nabilone for the treatment of pain in fibromyalgia. J. Pain.

[105] Maas A.I., Murray G., Henney III H., Kassem N., Legrand V., Mangelus M., Muizelaar J.-P., Stocchetti N., Knoller N. (2006). Efficacy and safety of dexanabinol in severe traumatic brain injury: Results of a phase III randomised, placebo-controlled, clinical trial. Lancet Neurol..

[106] Wyss-Coray T. (2016). Ageing, neurodegeneration and brain rejuvenation. Nature.

[107] Ransohoff R.M. (2016). How neuroinflammation contributes to neurodegeneration. Science.

[108] Allan S.M., Rothwell N.J. (2001). Cytokines and acute neurodegeneration. Nat. Rev. Neurosci..

[109] Mattson M.P. (2007). Calcium and neurodegeneration. Aging Cell.

[110] Knott A.B., Perkins G., Schwarzenbacher R., Bossy-Wetzel E. (2008). Mitochondrial fragmentation in neurodegeneration. Nat. Rev. Neurosci..

[111] Esposito G., Scuderi C., Savani C., Steardo Jr L., De Filippis D., Cottone P., Iuvone T., Cuomo V., Steardo L. (2007). Cannabidiol in vivo blunts β-amyloid induced neuroinflammation by suppressing IL-1β and iNOS expression. Br. J. Pharmacol..

[112] Mori M.A., Meyer E., Soares L.M., Milani H., Guimarães F.S., de Oliveira R.M.W. (2017). Cannabidiol reduces neuroinflammation and promotes neuroplasticity and functional recovery after brain ischemia. Prog. Neuro-Psychopharmacol. Biol. Psychiatry.

[113] Vallée A., Lecarpentier Y., Guillevin R., Vallée J.-N. (2017). Effects of cannabidiol interactions with Wnt/β-catenin pathway and PPARγ on oxidative stress and neuroinflammation in Alzheimer′s disease. Acta Biochim. Et Biophys. Sin..

[114] Lambert A.J., Brand M.D., Stuart J.A. (2009). Reactive oxygen species production by mitochondria. Mitochondrial DNA.

[115] Pisoschi A.M., Pop A. (2015). The role of antioxidants in the chemistry of oxidative stress: A review. Eur. J. Med. Chem..

[116] Liu Z., Zhou T., Ziegler A.C., Dimitrion P., Zuo L. (2017). Oxidative stress in neurodegenerative diseases: From molecular mechanisms to clinical applications. Oxidative Med. Cell. Longev..

[117] Rego A.C., Oliveira C.R. (2003). Mitochondrial Dysfunction and Reactive Oxygen Species in Excitotoxicity and Apoptosis: Implications for the Pathogenesis of Neurodegenerative Diseases. Neurochem. Res..

[118] Wang X., Michaelis E.K. (2010). Selective neuronal vulnerability to oxidative stress in the brain. Front. Aging Neurosci..

[119] Bih C.I., Chen T., Nunn A.V., Bazelot M., Dallas M., Whalley B.J. (2015). Molecular targets of cannabidiol in neurological disorders. Neurotherapeutics.

[120] Campos A.C., Fogaça M.V., Sonego A.B., Guimarães F.S. (2016). Cannabidiol, neuroprotection and neuropsychiatric disorders. Pharmacol. Res..

[121] Rosenberg E.C., Tsien R.W., Whalley B.J., Devinsky O. (2015). Cannabinoids and epilepsy. Neurotherapeutics.

[122] Pamplona F.A., da Silva L.R., Coan A.C. (2018). Potential clinical benefits of CBD-rich Cannabis extracts over purified CBD in treatment-resistant epilepsy: Observational data meta-analysis. Front. Neurol..

[123] Hussain S.A., Zhou R., Jacobson C., Weng J., Cheng E., Lay J., Hung P., Lerner J.T., Sankar R. (2015). Perceived efficacy of cannabidiol-enriched cannabis extracts for treatment of pediatric epilepsy: A potential role for infantile spasms and Lennox–Gastaut syndrome. Epilepsy Behav..

[124] Silvestro S., Mammana S., Cavalli E., Bramanti P., Mazzon E. (2019). Use of cannabidiol in the treatment of epilepsy: Efficacy and security in clinical trials. Molecules.

[125] Pazos M.R., Mohammed N., Lafuente H., Santos M., Martínez-Pinilla E., Moreno E., Valdizan E., Romero J., Pazos A., Franco R. (2013). Mechanisms of cannabidiol neuroprotection in hypoxic–ischemic newborn pigs: Role of 5HT1A and CB2 receptors. Neuropharmacology.

[126] Chen J.W., Borgelt L.M., Blackmer A.B. (2019). Cannabidiol: A new hope for patients with Dravet or Lennox-Gastaut syndromes. Ann. Pharmacother..

[127] Maggio N., Shavit Stein E., Segal M. (2018). Cannabidiol regulates long term potentiation following status epilepticus: Mediation by calcium stores and serotonin. Front. Mol. Neurosci..

[128] Fernández-Ruiz J., Romero J., Ramos J.A. (2015). Endocannabinoids and neurodegenerative disorders: Parkinson’s disease, Huntington’s chorea, Alzheimer’s disease, and others. Endocannabinoids.

[129] Nazıroğlu M., Övey I. (2015). Involvement of apoptosis and calcium accumulation through TRPV1 channels in neurobiology of epilepsy. Neuroscience.

[130] Lattanzi S., Brigo F., Trinka E., Zaccara G., Cagnetti C., Del Giovane C., Silvestrini M. (2018). Efficacy and safety of cannabidiol in epilepsy: A systematic review and meta-analysis. Drugs.

[131] Sveinbjornsdottir S. (2016). The clinical symptoms of Parkinson′s disease. J. Neurochem..

[132] Sonego A.B., Gomes F.V., Del Bel E.A., Guimaraes F.S. (2016). Cannabidiol attenuates haloperidol-induced catalepsy and c-Fos protein expression in the dorsolateral striatum via 5-HT1A receptors in mice. Behav. Brain Res..

[133] Peres F.F., Levin R., Suiama M.A., Diana M.C., Gouvêa D.A., Almeida V., Santos C.M., Lungato L., Zuardi A.W., Hallak J.E. (2016). Cannabidiol prevents motor and cognitive impairments induced by reserpine in rats. Front. Pharmacol..

[134] Peres F.F., Lima A.C., Hallak J.E., Crippa J.A., Silva R.H., Abílio V.C. (2018). Cannabidiol as a promising strategy to treat and prevent movement disorders?. Front. Pharmacol..

[135] Zuardi A.W., Crippa J., Hallak J.E.C., Pinto J., Chagas M.H.N., Rodrigues G., Dursun S., Tumas V. (2009). Cannabidiol for the treatment of psychosis in Parkinson’s disease. J. Psychopharmacol..

[136] Gomes F.V., Del Bel E.A., Guimarães F.S. (2013). Cannabidiol attenuates catalepsy induced by distinct pharmacological mechanisms via 5-HT1A receptor activation in mice. Prog. Neuro-Psychopharmacol. Biol. Psychiatry.

[137] Oeckl P., Hengerer B., Ferger B. (2014). G-protein coupled receptor 6 deficiency alters striatal dopamine and cAMP concentrations and reduces dyskinesia in a mouse model of Parkinson′s disease. Exp. Neurol..

[138] Congdon E.E., Sigurdsson E.M. (2018). Tau-targeting therapies for Alzheimer disease. Nat. Rev. Neurol..

[139] Esposito G., De Filippis D., Carnuccio R., Izzo A.A., Iuvone T. (2006). The marijuana component cannabidiol inhibits β-amyloid-induced tau protein hyperphosphorylation through Wnt/β-catenin pathway rescue in PC12 cells. J. Mol. Med..

[140] Casarejos M.J., Perucho J., Gomez A., Munoz M.P., Fernandez-Estevez M., Sagredo O., Fernandez Ruiz J., Guzman M., de Yebenes J.G., Mena M.A. (2013). Natural cannabinoids improve dopamine neurotransmission and tau and amyloid pathology in a mouse model of tauopathy. J. Alzheimer′s Dis..

[141] Martín-Moreno A.M., Reigada D., Ramírez B.G., Mechoulam R., Innamorato N., Cuadrado A., de Ceballos M.L. (2011). Cannabidiol and other cannabinoids reduce microglial activation in vitro and in vivo: Relevance to Alzheimer′s disease. Mol. Pharmacol..

[142] Janefjord E., Mååg J.L., Harvey B.S., Smid S.D. (2014). Cannabinoid effects on β amyloid fibril and aggregate formation, neuronal and microglial-activated neurotoxicity in vitro. Cell. Mol. Neurobiol..

[143] Watt G., Karl T. (2017). In vivo evidence for therapeutic properties of cannabidiol (CBD) for Alzheimer′s disease. Front. Pharmacol..

[144] Thameem Dheen S., Kaur C., Ling E.-A. (2007). Microglial activation and its implications in the brain diseases. Curr. Med. Chem..

[145] Maresz K., Carrier E.J., Ponomarev E.D., Hillard C.J., Dittel B.N. (2005). Modulation of the cannabinoid CB2 receptor in microglial cells in response to inflammatory stimuli. J. Neurochem..

[146] Hassan S., Eldeeb K., Millns P.J., Bennett A.J., Alexander S.P., Kendall D.A. (2014). Cannabidiol enhances microglial phagocytosis via transient receptor potential (TRP) channel activation. Br. J. Pharmacol..

[147] Manczak M., Sheiko T., Craigen W.J., Reddy P.H. (2013). Reduced VDAC1 protects against Alzheimer′s disease, mitochondria, and synaptic deficiencies. J. Alzheimer′s Dis..

[148] Leehey M.A., Liu Y., Hart F., Epstein C., Cook M., Sillau S., Klawitter J., Newman H., Sempio C., Forman L. (2020). Safety and tolerability of cannabidiol in Parkinson disease: An open label, dose-escalation study. Cannabis Cannabinoid Res..

[149] Patel A.D., Mazurkiewicz-Bełdzińska M., Chin R.F., Gil-Nagel A., Gunning B., Halford J.J., Mitchell W., Perry M.S., Thiele E.A., Weinstock A. (2021). Long-term safety and efficacy of add-on cannabidiol in patients with Lennox–Gastaut syndrome: Results of a long-term open-label extension trial. Epilepsia.

[150] Xu C., Chang T., Du Y., Yu C., Tan X., Li X. (2019). Pharmacokinetics of oral and intravenous cannabidiol and its antidepressant-like effects in chronic mild stress mouse model. Environ. Toxicol. Pharmacol..

[151] Shbiro L., Hen-Shoval D., Hazut N., Rapps K., Dar S., Zalsman G., Mechoulam R., Weller A., Shoval G. (2019). Effects of cannabidiol in males and females in two different rat models of depression. Physiol. Behav..

[152] Masataka N. (2019). Anxiolytic effects of repeated cannabidiol treatment in teenagers with social anxiety disorders. Front. Psychol..

[153] Giorgi V., Marotto D., Batticciotto A., Atzeni F., Bongiovanni S., Sarzi-Puttini P. (2021). Cannabis and autoimmunity: Possible mechanisms of action. ImmunoTargets Ther..

[154] Peball M., Seppi K., Krismer F., Knaus H.G., Spielberger S., Heim B., Ellmerer P., Werkmann M., Poewe W., Djamshidian A. (2022). Effects of Nabilone on Sleep Outcomes in Patients with Parkinson′s Disease: A Post-hoc Analysis of NMS-Nab Study. Mov. Disord. Clin. Pract..

[155] Wheless J.W., Dlugos D., Miller I., Oh D.A., Parikh N., Phillips S., Renfroe J.B., Roberts C.M., Saeed I., Sparagana S.P. (2019). Pharmacokinetics and tolerability of multiple doses of pharmaceutical-grade synthetic cannabidiol in pediatric patients with treatment-resistant epilepsy. CNS Drugs.

[156] Szaflarski J.P., Hernando K., Bebin E.M., Gaston T.E., Grayson L.E., Ampah S.B., Moreadith R. (2019). Higher cannabidiol plasma levels are associated with better seizure response following treatment with a pharmaceutical grade cannabidiol. Epilepsy Behav..

[157] Katchan V., David P., Shoenfeld Y. (2016). Cannabinoids and autoimmune diseases: A systematic review. Autoimmun. Rev..

[158] Furgiuele A., Cosentino M., Ferrari M., Marino F. (2021). Immunomodulatory potential of cannabidiol in multiple sclerosis: A systematic review. J. Neuroimmune Pharmacol..

[159] Keating G.M. (2017). Delta-9-tetrahydrocannabinol/cannabidiol oromucosal spray (Sativex®): A review in multiple sclerosis-related spasticity. Drugs.

[160] Mecha M., Feliú A., Iñigo P., Mestre L., Carrillo-Salinas F., Guaza C. (2013). Cannabidiol provides long-lasting protection against the deleterious effects of inflammation in a viral model of multiple sclerosis: A role for A2A receptors. Neurobiol. Dis..

[161] Kozela E., Lev N., Kaushansky N., Eilam R., Rimmerman N., Levy R., Ben-Nun A., Juknat A., Vogel Z. (2011). Cannabidiol inhibits pathogenic T cells, decreases spinal microglial activation and ameliorates multiple sclerosis-like disease in C57BL/6 mice. Br. J. Pharmacol..

[162] Liu Y., Wang X., Hu C.-A.A. (2017). Therapeutic potential of amino acids in inflammatory bowel disease. Nutrients.

[163] Ambrose T., Simmons A. (2019). Cannabis, cannabinoids, and the endocannabinoid system—is there therapeutic potential for inflammatory bowel disease?. J. Crohn′s Colitis.

[164] Hasenoehrl C., Storr M., Schicho R. (2017). Cannabinoids for treating inflammatory bowel diseases: Where are we and where do we go?. Expert Rev. Gastroenterol. Hepatol..

[165] Kienzl M., Storr M., Schicho R. (2020). Cannabinoids and opioids in the treatment of inflammatory bowel diseases. Clin. Transl. Gastroenterol..

[166] Martínez V., Iriondo De-Hond A., Borrelli F., Capasso R., Del Castillo M.D., Abalo R. (2020). Cannabidiol and other non-psychoactive cannabinoids for prevention and treatment of gastrointestinal disorders: Useful nutraceuticals?. Int. J. Mol. Sci..

[167] Vermeulen W., De Man J.G., Pelckmans P.A., De Winter B.Y. (2014). Neuroanatomy of lower gastrointestinal pain disorders. World J. Gastroenterol. WJG.

[168] Krohn R.M., Parsons S.A., Fichna J., Patel K.D., Yates R.M., Sharkey K.A., Storr M.A. (2016). Abnormal cannabidiol attenuates experimental colitis in mice, promotes wound healing and inhibits neutrophil recruitment. J. Inflamm..

[169] Couch D.G., Cook H., Ortori C., Barrett D., Lund J.N., O’Sullivan S.E. (2019). Palmitoylethanolamide and cannabidiol prevent inflammation-induced hyperpermeability of the human gut in vitro and in vivo—A randomized, placebo-controlled, double-blind controlled trial. Inflamm. Bowel Dis..

[170] Couch D.G., Tasker C., Theophilidou E., Lund J.N., O’Sullivan S.E. (2017). Cannabidiol and palmitoylethanolamide are anti-inflammatory in the acutely inflamed human colon. Clin. Sci..

[171] Pagano E., Capasso R., Piscitelli F., Romano B., Parisi O.A., Finizio S., Lauritano A., Marzo V.D., Izzo A.A., Borrelli F. (2016). An orally active cannabis extract with high content in cannabidiol attenuates chemically-induced intestinal inflammation and hypermotility in the mouse. Front. Pharmacol..

[172] Jamontt J., Molleman A., Pertwee R.G., Parsons M. (2010). The effects of Δ9-tetrahydrocannabinol and cannabidiol alone and in combination on damage, inflammation and in vitro motility disturbances in rat colitis. Br. J. Pharmacol..

[173] Borrelli F., Aviello G., Romano B., Orlando P., Capasso R., Maiello F., Guadagno F., Petrosino S., Capasso F., Di Marzo V. (2009). Cannabidiol, a safe and non-psychotropic ingredient of the marijuana plant Cannabis sativa, is protective in a murine model of colitis. J. Mol. Med..

[174] Khoury M., Cohen I., Bar-Sela G. (2022). “The Two Sides of the Same Coin”—Medical Cannabis, Cannabinoids and Immunity: Pros and Cons Explained. Pharmaceutics.

[175] Dörner T., Furie R. (2019). Novel paradigms in systemic lupus erythematosus. Lancet.

[176] Fava A., Petri M. (2019). Systemic lupus erythematosus: Diagnosis and clinical management. J. Autoimmun..

[177] Naftali T., Mechulam R., Marii A., Gabay G., Stein A., Bronshtain M., Laish I., Benjaminov F., Konikoff F.M. (2017). Low-dose cannabidiol is safe but not effective in the treatment for Crohn’s disease, a randomized controlled trial. Dig. Dis. Sci..

[178] Naftali T., Bar-Lev Schleider L., Almog S., Meiri D., Konikoff F.M. (2021). Oral CBD-rich cannabis induces clinical but not endoscopic response in patients with Crohn’s disease, a randomised controlled trial. J. Crohn′s Colitis.

[179] Irving P.M., Iqbal T., Nwokolo C., Subramanian S., Bloom S., Prasad N., Hart A., Murray C., Lindsay J.O., Taylor A. (2018). A randomized, double-blind, placebo-controlled, parallel-group, pilot study of cannabidiol-rich botanical extract in the symptomatic treatment of ulcerative colitis. Inflamm. Bowel Dis..

[180] Blake D.R., Robson P., Ho M., Jubb R.W., McCabe C.S. (2006). Preliminary assessment of the efficacy, tolerability and safety of a cannabis-based medicine (Sativex) in the treatment of pain caused by rheumatoid arthritis. Rheumatology.

[181] Hendricks O., Andersen T.E., Christiansen A.A., Primdahl J., Hauge E.M., Ellingsen T., Horsted T.I., Bachmann A.G., Loft A.G., Bojesen A.B. (2019). Efficacy and safety of cannabidiol followed by an open label add-on of tetrahydrocannabinol for the treatment of chronic pain in patients with rheumatoid arthritis or ankylosing spondylitis: Protocol for a multicentre, randomised, placebo-controlled study. BMJ Open.

[182] Kiriakidou M., Ching C.L. (2020). Systemic Lupus Erythematosus. Ann. Intern. Med..

[183] Navarini L., Bisogno T., Mozetic P., Piscitelli F., Margiotta D.P.E., Basta F., Afeltra A., Maccarrone M. (2018). Endocannabinoid system in systemic lupus erythematosus: First evidence for a deranged 2-arachidonoylglycerol metabolism. Int. J. Biochem. Cell Biol..

[184] Rahaman O., Bhattacharya R., Liu C.S.C., Raychaudhuri D., Ghosh A.R., Bandopadhyay P., Pal S., Goswami R.P., Sircar G., Ghosh P. (2019). Cutting edge: Dysregulated endocannabinoid-rheostat for plasmacytoid dendritic cell activation in a systemic lupus endophenotype. J. Immunol..

[185] Henriquez J.E., Crawford R.B., Kaminski N.E. (2019). Suppression of CpG-ODN-mediated IFNα and TNFα response in human plasmacytoid dendritic cells (pDC) by cannabinoid receptor 2 (CB2)-specific agonists. Toxicol. Appl. Pharmacol..

[186] Maddukuri S., Patel J., Diaz D.A., Chen K.L., Wysocka M., Bax C., Li Y., Ravishankar A., Grinnell M., Zeidi M. (2022). Cannabinoid type 2 receptor (CB2R) distribution in dermatomyositis skin and peripheral blood mononuclear cells (PBMCs) and in vivo effects of LenabasumTM. Arthritis Res. Ther..

[187] Aletaha D., Smolen J.S. (2018). Diagnosis and management of rheumatoid arthritis: A review. Jama.

[188] Burmester G.R., Pope J.E. (2017). Novel treatment strategies in rheumatoid arthritis. Lancet.

[189] Lowin T., Schneider M., Pongratz G. (2019). Joints for joints: Cannabinoids in the treatment of rheumatoid arthritis. Curr. Opin. Rheumatol..

[190] Gui H., Liu X., Wang Z.-W., He D.-Y., Su D.-F., Dai S.-M. (2014). Expression of cannabinoid receptor 2 and its inhibitory effects on synovial fibroblasts in rheumatoid arthritis. Rheumatology.

[191] Gui H., Liu X., Liu L.-R., Su D.-F., Dai S.-M. (2015). Activation of cannabinoid receptor 2 attenuates synovitis and joint distruction in collagen-induced arthritis. Immunobiology.

[192] Lowin T., Tingting R., Zurmahr J., Classen T., Schneider M., Pongratz G. (2020). Cannabidiol (CBD): A killer for inflammatory rheumatoid arthritis synovial fibroblasts. Cell Death Dis..

[193] Campanati A., Marani A., Martina E., Diotallevi F., Radi G., Offidani A. (2021). Psoriasis as an immune-mediated and inflammatory systemic disease: From pathophysiology to novel therapeutic approaches. Biomedicines.

[194] Dopytalska K., Ciechanowicz P., Wiszniewski K., Szymańska E., Walecka I. (2021). The role of epigenetic factors in psoriasis. Int. J. Mol. Sci..

[195] Carvalho A.L., Hedrich C.M. (2021). The molecular pathophysiology of psoriatic arthritis—the complex interplay between genetic predisposition, epigenetics factors, and the microbiome. Front. Mol. Biosci..

[196] Sheriff T., Lin M.J., Dubin D., Khorasani H. (2020). The potential role of cannabinoids in dermatology. J. Dermatol. Treat..

[197] Ramot Y., Sugawara K., Zákány N., Toth B.I., Bíró T., Paus R. (2013). A novel control of human keratin expression: Cannabinoid receptor 1-mediated signaling down-regulates the expression of keratins K6 and K16 in human keratinocytes in vitro and in situ. PeerJ.

[198] Wilkinson J.D., Williamson E.M. (2007). Cannabinoids inhibit human keratinocyte proliferation through a non-CB1/CB2 mechanism and have a potential therapeutic value in the treatment of psoriasis. J. Dermatol. Sci..

[199] Norooznezhad A.H., Norooznezhad F. (2017). Cannabinoids: Possible agents for treatment of psoriasis via suppression of angiogenesis and inflammation. Med. Hypotheses.

[200] Scheau C., Badarau I.A., Mihai L.-G., Scheau A.-E., Costache D.O., Constantin C., Calina D., Caruntu C., Costache R.S., Caruntu A. (2020). Cannabinoids in the pathophysiology of skin inflammation. Molecules.

[201] Stella A., Palmieri B., Laurino C., Vadalà M. (2019). A therapeutic effect of cbd-enriched ointment in inflammatory skin diseases and cutaneous scars. La Clin. Ter..

[202] Sinai A., Turner Z., Baruch Y. (2020). Cannabis-based extracts and topical formulations for use in skin disorders. U.S. Patent.

[203] Guzmán M. (2003). Cannabinoids: Potential anticancer agents. Nat. Rev. Cancer.

[204] Aggarwal S.K., Carter G.T., Sullivan M.D., ZumBrunnen C., Morrill R., Mayer J.D. (2009). Medicinal use of cannabis in the United States: Historical perspectives, current trends, and future directions. J. Opioid Manag..

[205] Reuter S.E., Martin J.H. (2016). Pharmacokinetics of cannabis in cancer cachexia-anorexia syndrome. Clin. Pharmacokinet..

[206] Borgelt L.M., Franson K.L., Nussbaum A.M., Wang G.S. (2013). The P harmacologic and C linical E ffects of M edical C annabis. Pharmacother. J. Hum. Pharmacol. Drug Ther..

[207] Barnes M.P. (2006). Sativex®: Clinical efficacy and tolerability in the treatment of symptoms of multiple sclerosis and neuropathic pain. Expert Opin. Pharmacother..

[208] Blake A., Wan B.A., Malek L., DeAngelis C., Diaz P., Lao N., O’Hearn S. (2017). A selective review of medical cannabis in cancer pain management. Ann. Palliat. Med..

[209] Velasco G., Sánchez C., Guzmán M. (2012). Towards the use of cannabinoids as antitumour agents. Nat. Rev. Cancer.

[210] Chakravarti B., Ravi J., Ganju R.K. (2014). Cannabinoids as therapeutic agents in cancer: Current status and future implications. Oncotarget.

[211] Velasco G., Sánchez C., Guzmán M. (2016). Anticancer mechanisms of cannabinoids. Curr. Oncol..

[212] Pisanti S., Malfitano A.M., Ciaglia E., Lamberti A., Ranieri R., Cuomo G., Abate M., Faggiana G., Proto M.C., Fiore D. (2017). Cannabidiol: State of the art and new challenges for therapeutic applications. Pharmacol. Ther..

[213] Kim J.L., Kim B.R., Kim D.Y., Jeong Y.A., Jeong S., Na Y.J., Park S.H., Yun H.K., Jo M.J., Kim B.G. (2019). Cannabidiol Enhances the Therapeutic Effects of TRAIL by Upregulating DR5 in Colorectal Cancer. Cancers.

[214] Lukhele S.T., Motadi L.R. (2016). Cannabidiol rather than Cannabis sativa extracts inhibit cell growth and induce apoptosis in cervical cancer cells. BMC Complement. Altern. Med..

[215] Elbaz M., Nasser M.W., Ravi J., Wani N.A., Ahirwar D.K., Zhao H., Oghumu S., Satoskar A.R., Shilo K., Carson III W.E. (2015). Modulation of the tumor microenvironment and inhibition of EGF/EGFR pathway: Novel anti-tumor mechanisms of Cannabidiol in breast cancer. Mol. Oncol..

[216] Jeong S., Jo M.J., Yun H.K., Kim D.Y., Kim B.R., Kim J.L., Park S.H., Na Y.J., Jeong Y.A., Kim B.G. (2019). Cannabidiol promotes apoptosis via regulation of XIAP/Smac in gastric cancer. Cell Death Dis..

[217] Huang T., Xu T., Wang Y., Zhou Y., Yu D., Wang Z., He L., Chen Z., Zhang Y., Davidson D. (2021). Cannabidiol inhibits human glioma by induction of lethal mitophagy through activating TRPV4. Autophagy.

[218] Kosgodage U.S., Mould R., Henley A.B., Nunn A.V., Guy G.W., Thomas E.L., Inal J.M., Bell J.D., Lange S. (2018). Cannabidiol (CBD) is a novel inhibitor for exosome and microvesicle (EMV) release in cancer. Front. Pharmacol..

[219] Schoeman R., Beukes N., Frost C. (2020). Cannabinoid combination induces cytoplasmic vacuolation in MCF-7 breast cancer cells. Molecules.

[220] Milian L., Mata M., Alcacer J., Oliver M., Sancho-Tello M., Martín de Llano J.J., Camps C., Galbis J., Carretero J., Carda C. (2020). Cannabinoid receptor expression in non-small cell lung cancer. Effectiveness of tetrahydrocannabinol and cannabidiol inhibiting cell proliferation and epithelial-mesenchymal transition in vitro. PLoS ONE.

[221] Nabissi M., Morelli M.B., Offidani M., Amantini C., Gentili S., Soriani A., Cardinali C., Leoni P., Santoni G. (2016). Cannabinoids synergize with carfilzomib, reducing multiple myeloma cells viability and migration. Oncotarget.

[222] Jeong S., Kim B.G., Kim D.Y., Kim B.R., Kim J.L., Park S.H., Na Y.J., Jo M.J., Yun H.K., Jeong Y.A. (2019). Cannabidiol overcomes oxaliplatin resistance by enhancing NOS3-and SOD2-Induced autophagy in human colorectal cancer cells. Cancers.

[223] Deng L., Ng L., Ozawa T., Stella N. (2017). Quantitative analyses of synergistic responses between cannabidiol and DNA-damaging agents on the proliferation and viability of glioblastoma and neural progenitor cells in culture. J. Pharmacol. Exp. Ther..

[224] López-Valero I., Saiz-Ladera C., Torres S., Hernández-Tiedra S., García-Taboada E., Rodríguez-Fornés F., Barba M., Dávila D., Salvador-Tormo N., Guzmán M. (2018). Targeting glioma initiating cells with a combined therapy of cannabinoids and temozolomide. Biochem. Pharmacol..

[225] Fraguas-Sánchez A., Fernández-Carballido A., Simancas-Herbada R., Martin-Sabroso C., Torres-Suárez A. (2020). CBD loaded microparticles as a potential formulation to improve paclitaxel and doxorubicin-based chemotherapy in breast cancer. Int. J. Pharm..

[226] Twelves C., Sabel M., Checketts D., Miller S., Tayo B., Jove M., Brazil L., Short S.C. (2021). A phase 1b randomised, placebo-controlled trial of nabiximols cannabinoid oromucosal spray with temozolomide in patients with recurrent glioblastoma. Br. J. Cancer.

[227] Bar-Sela G., Cohen I., Campisi-Pinto S., Lewitus G.M., Oz-Ari L., Jehassi A., Peer A., Turgeman I., Vernicova O., Berman P. (2020). Cannabis consumption used by cancer patients during immunotherapy correlates with poor clinical outcome. Cancers.

[228] Chesney E., Oliver D., Green A., Sovi S., Wilson J., Englund A., Freeman T.P., McGuire P. (2020). Adverse effects of cannabidiol: A systematic review and meta-analysis of randomized clinical trials. Neuropsychopharmacology.

